# Automated SEM-based nanoparticle metrology for materials characterization *via* segmentation and robust scale-bar recognition

**DOI:** 10.1039/d6ra02763f

**Published:** 2026-08-05

**Authors:** Xueyi Huang, Yuzhong Yin, Jinghao Hu, Wenxiao Yu, Liang Fang, Jian Liu, Dongdong Li

**Affiliations:** a School of Computer Science and Information Engineering, Hefei University of Technology Hefei 230009 China jianliu@hfut.edu.cn; b School of Materials Science and Engineering, Hefei University of Technology Hefei 230009 China ddli@hfut.edu.cn; c School of Mechanical Engineering, Hefei University of Technology Hefei 230009 China

## Abstract

Quantitative nanoparticle metrology is essential for establishing structure–property relationships in catalysis, drug delivery, optoelectronic materials, battery materials, and synthesis optimization. Scanning electron microscopy (SEM) provides direct access to particle morphology, but high-throughput and reproducible analysis remains limited by ambiguous particle boundaries, particle agglomeration, and unreliable scale calibration. Here, we report an automated SEM-based nanoparticle metrology workflow that converts raw SEM images into scale-calibrated particle-size distributions and morphology statistics. The framework integrates Multi-scale U-shaped Kolmogorov–Arnold Network (MU-KAN) segmentation with YOLOv11-based scale-bar localization and optical character recognition-assisted scale annotation parsing. On the public NanoSEM-464 and NanoSEM-1707 benchmarks, MU-KAN achieves IoU values of 0.9296 and 0.8243 and F1-scores of 0.9630 and 0.9029, respectively. The scale recovery module gives a mean relative error of 3.8604% on a 370-image SEM scale-bar dataset. The resulting workflow provides particle area, equivalent circular diameter, circularity, and complementary shape descriptors, enabling automated, reproducible, and physically meaningful nanoparticle characterization for materials research.

## Abstract 2

Quantitative nanoparticle metrology is essential for establishing structure–property relationships in catalysis, drug delivery, optoelectronic materials, battery materials, and synthesis optimization.

## Graphical abstract

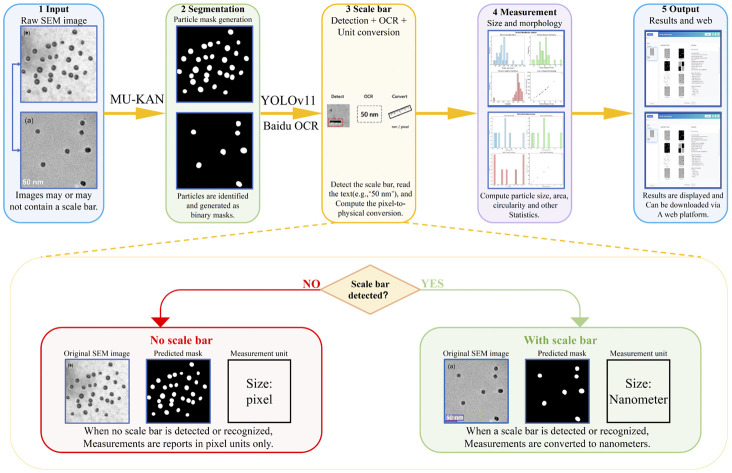

## 1 Introduction

Nanoparticle size distributions and morphological characteristics are critical microscopic parameters that govern macroscopic performance in catalysis,^1^ drug delivery,^2^ and optoelectronic materials.^3^ Reliable nanoparticle metrology is therefore central to materials design, synthesis optimization, structure–property analysis, and quality control.

Scanning electron microscopy (SEM) is widely used in chemistry and materials research because it offers high spatial resolution and direct observation of particle morphology.^4^ Although UV-visible spectroscopy, X-ray diffraction, and laser diffraction can estimate particle size, these methods are indirect and may introduce systematic deviations under complex sample conditions. Quantitative SEM analysis, however, still commonly depends on manual or semi-manual measurement, which is labor-intensive, subjective, and difficult to scale to high-throughput characterization and statistically robust reporting.^5^

Automated SEM-based nanoparticle metrology is constrained by two coupled bottlenecks: reliable extraction of particle boundaries and accurate scale calibration for physical-unit conversion. Boundary errors propagate directly into diameter, area, circularity, and other morphology descriptors, while scale-bar errors bias every physical measurement. Practical SEM images further contain weak foreground–background contrast, nonuniform illumination, acquisition noise, irregular shapes, broad size distributions, and particle overlap or agglomeration. These challenges have been widely reported in SEM image analysis studies, including machine vision-based nanoparticle characterization methods,^6^ deep learning-based segmentation frameworks for SEM images,^7^ and classical clustering-based and watershed-based approaches for granular object segmentation.^8^

Earlier pipelines mainly relied on traditional image processing, including thresholding, morphological operations,^9^ distance transforms, and watershed-based separation.^10^ Such rule-driven approaches can be effective under clean imaging conditions but often degrade under low contrast, noise, and overlap, and typically require extensive parameter tuning across microscopes and samples, which restricts generalization and reproducibility. These limitations motivate learning-based approaches that can better adapt to diverse SEM imaging distributions.^11^ Recent advances in image-driven machine learning and deep learning have significantly improved robustness and performance in microscopy image analysis.^12^ Supervised deep learning has been increasingly applied to nanoparticle SEM segmentation, and U-Net–style encoder–decoder architectures remain widely adopted due to their effective spatial – semantic fusion. Recent studies have explored advanced data augmentation strategies to improve model robustness. Semantic-similarity-based GAN augmentation has been proposed for small-sample image learning,^13^ while GAN-based augmentation has demonstrated effectiveness in agricultural image segmentation,^14^ OCT image analysis,^15^ and dermoscopy image processing.^16^ Nevertheless, reliably segmenting small/low-contrast particles in cluttered backgrounds and maintaining boundary continuity in dense agglomeration remain persistent failure modes,^17^ which directly affect downstream metrology fidelity even when pixel-level metrics appear competitive. Beyond segmentation, practical SEM metrology additionally requires pixel-to-physical calibration through scale-bar recovery. Existing strategies include heuristic region selection around annotation text, segmentation-based scale extraction, and direct regression approaches, but SEM scale annotations vary substantially in position,^18^ contrast polarity (white-on-black or black-on-white), resolution, and style across microscope manufacturers and acquisition settings. As a result, scale-bar recognition can become an independent bottleneck that prevents end-to-end automation and consistent physical-size reporting.^19^ Meanwhile, recent advances in deep learning architectures, particularly Kolmogorov–Arnold Networks (KANs), have attracted increasing attention because of their nonlinear functional representation mechanism and their potential applications in image segmentation tasks.^20^

To address these coupled materials-characterization needs, this work establishes an automated SEM metrology framework whose contributions are organized around physical nanoparticle measurement: (i) an automated workflow that converts raw SEM images into particle-size distributions and morphology statistics; (ii) robust scale-bar recovery for pixel-to-physical calibration; (iii) MU-KAN segmentation for reliable particle masks under diverse SEM conditions; (iv) a publicly released 370-image scale-bar benchmark for calibration assessment; and (v) a web implementation for reproducible, high-throughput nanoparticle characterization. In this workflow, the segmentation architecture and scale-recognition components serve as technical modules that enable quantitative materials metrology rather than as stand-alone algorithmic endpoints.

## 2 Materials and methods

### 2.1 SEM datasets and nanoparticle metrology task

#### 2.1.1 SEM datasets and split protocol

Two public SEM nanoparticle segmentation benchmarks were used for evaluation: NanoSEM-464 (ref. 21) and NanoSEM-1707.^22^ NanoSEM-464 contains 464 SEM images with pixel-level annotations and was split into training and validation sets at a ratio of 8 : 2 (371/93). NanoSEM-1707 contains 1707 SEM images and was divided into training/validation/test sets at a ratio of 8 : 1 : 1, providing a larger dataset and greater variability in particle morphology, particle density, and imaging conditions, thereby enabling a more comprehensive evaluation of model robustness and cross-dataset generalization. Unless otherwise specified, images were resized to 256 × 256 before being fed into the network to standardize input resolution and computational cost. The selection of this resolution was further validated through an input-resolution ablation study (SI Section S8 and Table S7), which showed that 256 × 256 achieved the best overall segmentation performance among the tested resolutions. To ensure reproducibility and avoid potential information leakage, the NanoSEM-464 dataset was randomly partitioned into training and test subsets using a fixed random seed of 2981. Data augmentation operations were applied exclusively to the training subset after dataset partitioning, while the test subset was reserved solely for performance evaluation and was not involved in model training, model selection, or hyperparameter tuning. For NanoSEM-1707, the predefined training, validation, and test partitions provided with the public dataset were directly adopted without any manual reassignment of samples. Consequently, all reported results were obtained using mutually exclusive subsets throughout the entire experimental process.

#### 2.1.2 Scale-bar subset construction and ground-truth definition

To enable physical-size quantification, a scale-bar-available subset was constructed from NanoSEM-464 by excluding images without scale bars or with severely degraded scale annotations. The resulting subset contains 370 SEM images. For each scale bar, ground-truth pixel length was defined using the center points of the two long sides of the rectangular scale bar; the absolute difference between their horizontal coordinates was recorded as the scale-bar pixel length. This definition is consistent with common SEM scale-bar designs, where scale information is encoded by the horizontal extent of a rectangular scale bar, as also adopted in previous SEM scale-bar extraction studies.^23^

### 2.2 Overall automated metrology workflow

#### 2.2.1 Workflow definition and metrology sequence

**Fig. 1:**
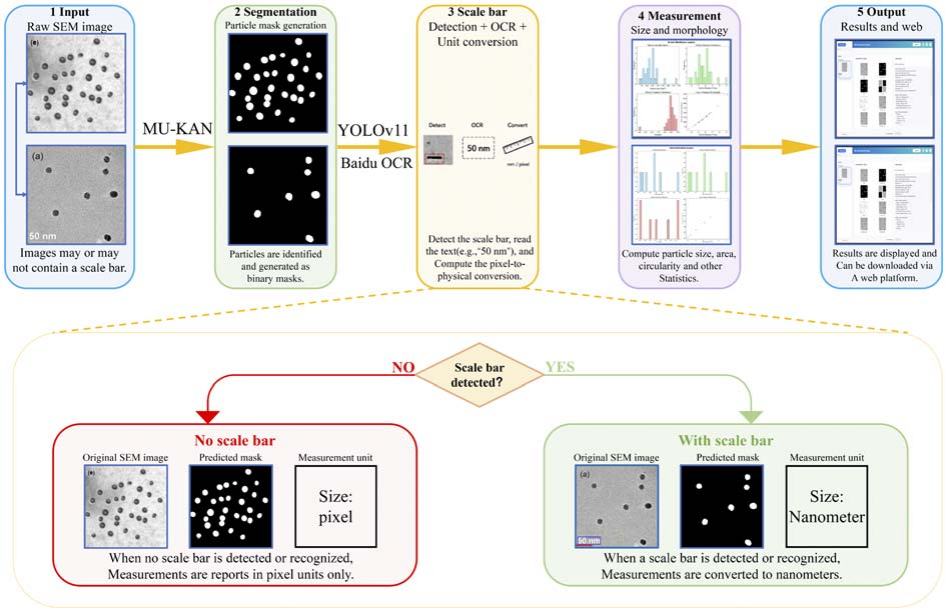
Schematic of the proposed SEM-based nanoparticle metrology framework, including segmentation, scale-bar calibration, and quantitative measurement of particle size and morphology. The top panel shows the overall workflow, and the bottom panel illustrates the scale-bar detection and unit conversion logic.

The proposed study targets automated nanoparticle metrology from SEM images, which requires two coupled capabilities: (i) accurate particle segmentation under low contrast, noise, and agglomeration (where low contrast refers to limited grayscale separation between particle boundaries and the surrounding background, noise refers to acquisition-related intensity fluctuations and textured background artifacts, and agglomeration refers to densely distributed particles with adjacent but still visually distinguishable boundaries; representative examples are shown in SI Fig. S10, which illustrates weak particle-background contrast and background interference, and SI Fig. S14, which illustrates mild-to-moderate particle agglomeration with reduced boundary separability),^24^ and (ii) reliable pixel-to-physical calibration through automated scale-bar recovery. The complete workflow, including SEM image input, segmentation inference, scale recovery, and quantitative size statistics, is summarized in Fig. 1. This workflow definition is used consistently throughout the paper to connect algorithmic components to the evaluation sections reported later (segmentation metrics and calibration error).^25^ In this study, mild-to-moderate agglomeration denotes particles for which at least part of the inter-particle boundary remains visually distinguishable. In contrast, extremely dense agglomeration or severe overlap denotes cases where neighboring particles are in direct contact and the projected boundary cannot be reliably resolved; such cases are treated as a limitation rather than a fully solved scenario.

### 2.3 Particle segmentation module

#### 2.3.1 Role of particle segmentation in the metrology workflow

**Fig. 2:**
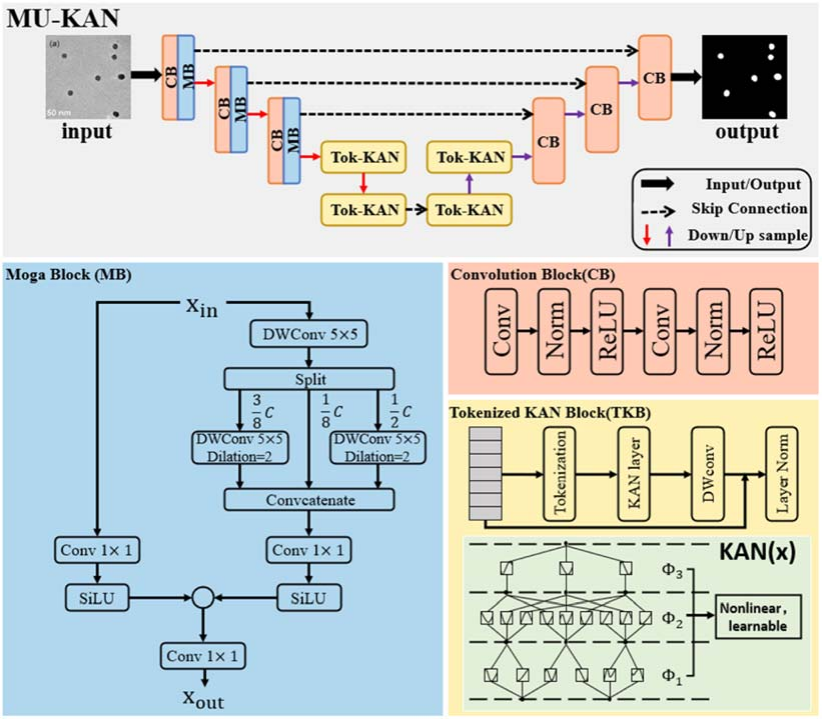
Architecture of the proposed MU-KAN network.

The particle segmentation module supports reliable identification of small or low-contrast nanoparticles under complex SEM backgrounds.^26^ Its architecture is illustrated in Fig. 2. Similar to semantic segmentation workflows widely adopted in materials image analysis,^27^ the resulting masks enable contour-based morphology descriptors for quantitative microstructure and particle characterization,^28^ as well as particle-size statistics and distribution analysis.^29^ The calibration module supplies pixel-to-physical conversion to complete quantitative materials characterization.

#### 2.3.2 MU-KAN architecture

MU-KAN follows a U-Net-style encoder–decoder framework with multi-level skip connections for spatial–semantic fusion. Two core designs are integrated:

(1) MB-based multi-scale encoding: multi-scale feature interaction is introduced in the encoder to facilitate feature extraction across particle sizes and mitigate the influence of size-distribution variations. This component targets missed detections of small particles and incomplete boundaries in low-contrast regions.

(2) KAN-driven nonlinear bottleneck: a tokenized KAN bottleneck is incorporated near the feature bottleneck to provide nonlinear mapping capacity and stabilize boundary representation under nonuniform grayscale and noise. This component targets SEM-specific artifacts such as blurred edges and local intensity fluctuations.^30^

The overall block organization and module placement are shown in Fig. 2.

### 2.4 Scale-bar recognition and pixel-to-physical calibration

#### 2.4.1 Module positioning in the metrology workflow

Scale-bar recognition is required to convert pixel-level measurements into physical units and is evaluated independently in the results section (relative error and prediction-ground truth correlation). Within the end-to-end workflow (Fig. 1), the calibration module takes raw SEM images as input, outputs scale-bar pixel length and scale text values, and provides the conversion factor used by the measurement module.^31^

#### 2.4.2 Scale-bar localization (YOLOv11)

Scale bars were localized using a YOLOv11-based detector, which identifies the approximate scale-bar region in SEM images. Rather than directly using the width of the detected bounding box as the scale-bar length, the detected region was further processed to extract the actual scale-bar segment.^32^ Specifically, grayscale conversion, threshold segmentation, and morphological operations were applied within the detected region to isolate the scale-bar structure. A minimum-area rotated bounding rectangle was then fitted to the extracted scale-bar segment. The scale-bar pixel length *L*_px_was defined as:

1 *L*_px_ = max(*W*_rot_, *H*_rot_)

where *W*_rot_ and *H*_rot_ are the width and height of the minimum-area rotated rectangle, respectively. This strategy reduces potential measurement bias caused by localization offsets, surrounding annotations, and slight scale-bar rotations.

Calibration accuracy was evaluated using the relative error formula:2
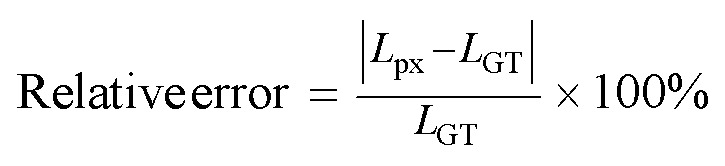
where *L*_px_, *L*_GT_ is the ground-truth pixel length of the scale bar. This approach provides a unified calibration strategy across different scale lengths and imaging conditions.

#### 2.4.3 Scale-text parsing (OCR) and unit handling

**Fig. 3:**
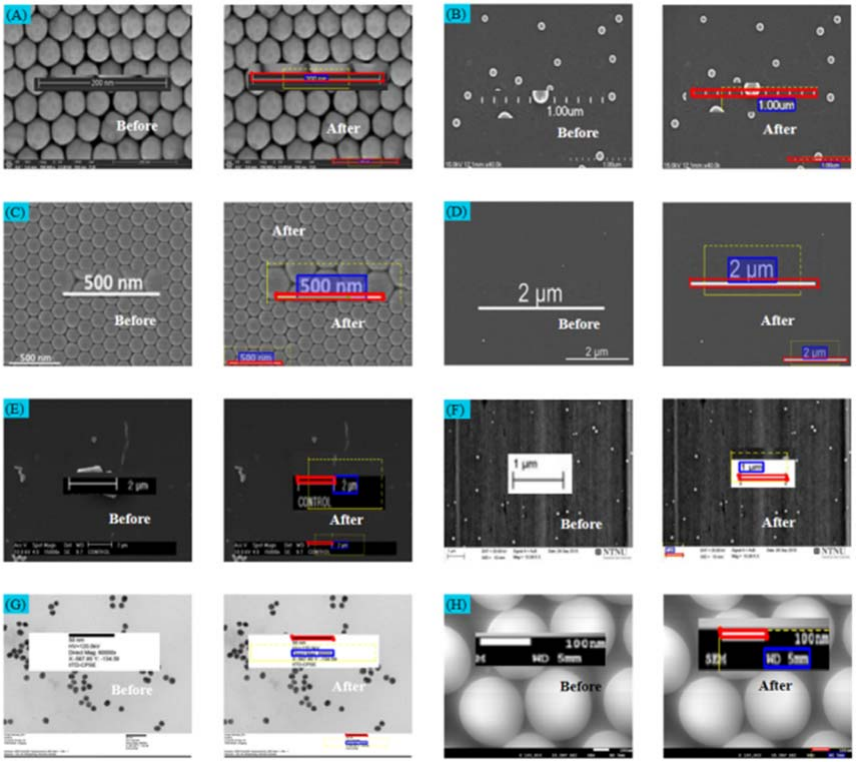
Scale-bar recognition results across diverse SEM images, including six representative successful cases (A–F) and two challenging failure cases (G–H). “Before” shows the original image and “After” shows the recognition overlay.

The numerical value and unit (*e.g.*, nm and µm) adjacent to the scale bar were parsed using Baidu OCR, a commercial OCR service developed by Baidu, Inc. (China). Baidu OCR was adopted as an off-the-shelf text-recognition module because of its robust recognition performance for mixed numerical-text annotations and measurement units commonly encountered in SEM images. The OCR outputs were further post-processed to robustly extract the numerical magnitude and unit under diverse annotation styles. A comparison with other commonly used OCR methods (Tesseract OCR and EasyOCR) is provided in SI Section S9, where Baidu OCR achieved the highest recognition accuracy on the 370-image scale-bar dataset used in this study. As illustrated by six representative cases in Fig. 3(A–F), the proposed scale-bar recognition module is capable of handling substantial variations in scale-bar appearance, including different contrast polarities (bright text on dark backgrounds and dark text on bright backgrounds), heterogeneous backgrounds (dense particle textures *versus* smooth or noisy fields), and diverse layout patterns (scale bars embedded in corners, placed near the image center, or floating within the field of view). In Fig. 3, the “Before” panels correspond to the original SEM images, while the “After” panels show the recognized scale-bar region and the parsed scale text overlaid on the image. These examples demonstrate that both the scale-bar geometry and its associated textual annotation can be reliably identified across different positions, sizes, and units, providing a stable basis for subsequent pixel-to-physical calibration and quantitative size measurement.

The proposed scale-bar recognition module was further evaluated on a self-built dataset containing 370 SEM images. With BaiduOCR, 365 images were correctly recognized, achieving an accuracy of 98.64%, while 5 images failed. These failure cases can be broadly divided into two categories: occlusion/fusion cases and abnormal-layout cases. Representative examples are shown in Fig. 3(G–H). Specifically, Fig. 3(G) belongs to the abnormal-layout category, where the scale-bar annotation adopts an unconventional arrangement, reducing the reliability of OCR-based text recognition. Fig. 3(H) belongs to the occlusion/fusion category, where the scale-bar annotation is embedded within or partially overlapped with surrounding image structures, interfering with scale-bar localization. These challenging cases account for the remaining recognition errors and reveal the current limitations of the scale-bar recognition module under complex annotation layouts and strong visual interference.

### 2.5 Particle morphology descriptors and statistical outputs

#### 2.5.1 Pixel-to-physical conversion

Given the recovered scale-bar pixel length *L*_px_ and the physical length *L*_phy_ parsed from the scale annotation, the pixel-to-physical conversion factor is computed as:

3 *k* = *L*_phy_/*L*_px_

where *k* denotes the physical size per pixel (*e.g.*, nm per pixel or µm per pixel).

#### 2.5.2 Particle morphology descriptors

For each connected particle region in the predicted segmentation mask, geometric descriptors were computed, including area, perimeter, equivalent circular diameter, and circularity. In particular:

Pixel area *A* is converted to physical area as:

4 *A*_phy_ = *k*^2^·*A*

where *A*_phy_ denotes the physical area of the particle region and *A* is the corresponding area in pixel units.

Equivalent circular diameter was computed as:5
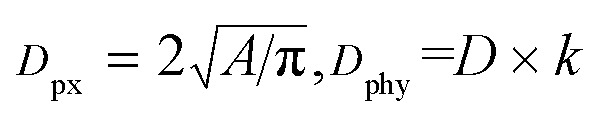
where *D*_px_ and *D*_phy_ denote the equivalent circular diameter in pixel units and physical units, respectively.

Circularity is defined as:

6 *C* = 4π·*A*/*P*^2^

where *P* denotes the perimeter of the particle region in pixel units.

The system then outputs particle-size distributions (area/diameter/circularity histograms) and summary statistics as part of the workflow shown in Fig. 1, supporting direct SEM metrology rather than segmentation-only outputs.

### 2.6 Training and evaluation protocol

#### 2.6.1 Training configuration and optimization

All experiments were implemented in PyTorch and trained on an NVIDIA RTX 4090 GPU. The Adam optimizer was used for network optimization with a weight decay of 1 × 10^−4^, which is a widely adopted regularization strategy in deep learning-based image segmentation. Weight decay acts as an L2 regularization term that penalizes large model weights, thereby reducing overfitting and improving generalization performance.

(1) For NanoSEM-464, the models were trained for 200 epochs with a batch size of 8 and an initial learning rate of 3 × 10^−4^.

(2) For NanoSEM-1707, the models were trained for 300 epochs with a batch size of 16 and an initial learning rate of 4 × 10^−4^. The number of epochs was selected based on convergence behavior to ensure stable training and fully converged validation performance.

(3) Binary cross-entropy (BCE) loss was adopted as the default optimization objective for pixel-wise foreground–background segmentation. For KAN-related parameters, a smaller learning rate of 1 × 10^−5^ was applied to improve optimization stability. A cosine annealing learning-rate scheduler (CosineAnnealingLR) was used, with the minimum learning rate set to 1 × 10^−5^ to facilitate stable convergence.

(4) A fixed random seed of 2981 was used throughout all experiments to ensure reproducibility of dataset partitioning, model initialization, and training procedures. This value is widely used as an arbitrary but fixed constant in deep learning research to guarantee reproducibility rather than affecting model performance. Data augmentation was applied exclusively to the training subset after dataset partitioning.

In addition, hyperparameter optimization was conducted using the Optuna framework, where learning rate, batch size, weight decay, and scheduler settings were systematically explored based on validation Intersection over Union (IoU). To further assess robustness, five-fold cross-validation was additionally performed on the NanoSEM-464 dataset. Detailed optimization and validation results are provided in SI Section S7.

#### 2.6.2 Evaluation metrics

Segmentation performance was evaluated using IoU, F1-score, accuracy, precision, and binary cross-entropy (BCE) loss, consistent with the quantitative comparisons reported later. Let TP, FP, TN, and FN denote true positives, false positives, true negatives, and false negatives at the pixel level, respectively. *N* denotes the total number of pixels in the evaluation image. A small smoothing constant *ε* was used to avoid division-by-zero. For the BCE loss formulation, *y* and *p* denote the ground-truth label and predicted probability, respectively.

These metrics provide complementary views of boundary fidelity, region overlap, and classification reliability for SEM nanoparticle segmentation.7
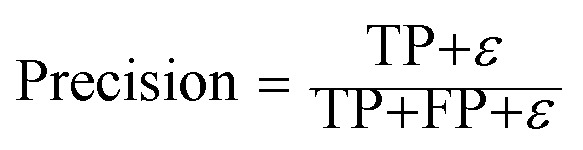
8
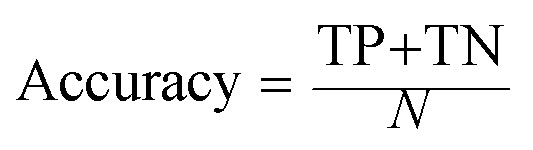
9
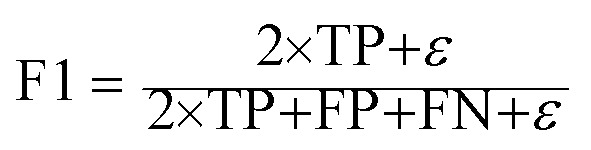
10
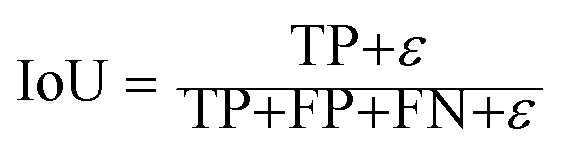
11
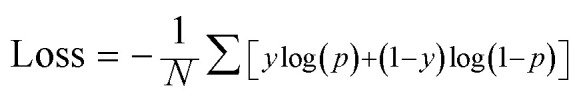


## 3 Results and discussion

### 3.1 End-to-end nanoparticle measurement workflow and representative outputs

#### 3.1.1 Integrated SEM-to-statistics workflow

**Fig. 4:**
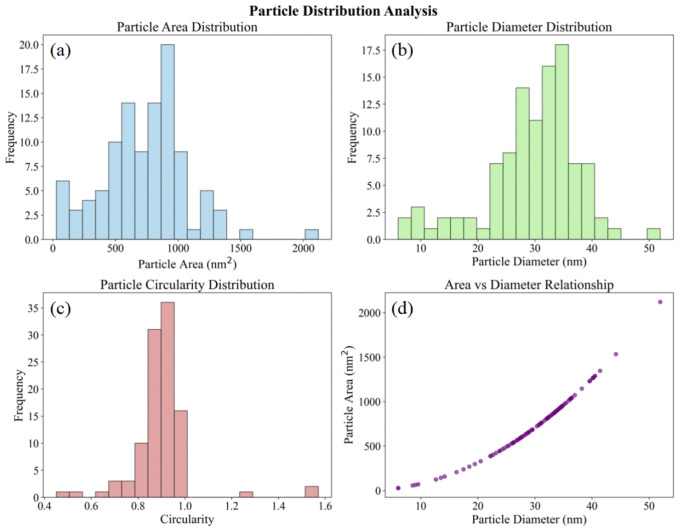
Examples of statistical analysis results of (a) particle area distributions, (b) equivalent circular diameter distributions, (c) circularity distributions, and (d) area–diameter scatter plots for internal consistency analysis.

A central objective of this work is to establish an end-to-end materials characterization workflow that transforms SEM images into quantitative particle measurements. As illustrated in Fig. 1, the framework generates particle masks and contours, performs pixel-to-physical calibration using the scale-bar recognition module, and extracts particle-level morphology descriptors and statistical distributions. This integrated sequence converts raw SEM images into physically meaningful nanoparticle characterization outputs rather than segmentation masks alone Fig. 4.

#### 3.1.2 Representative statistical outputs and web implementation

**Fig. 5:**
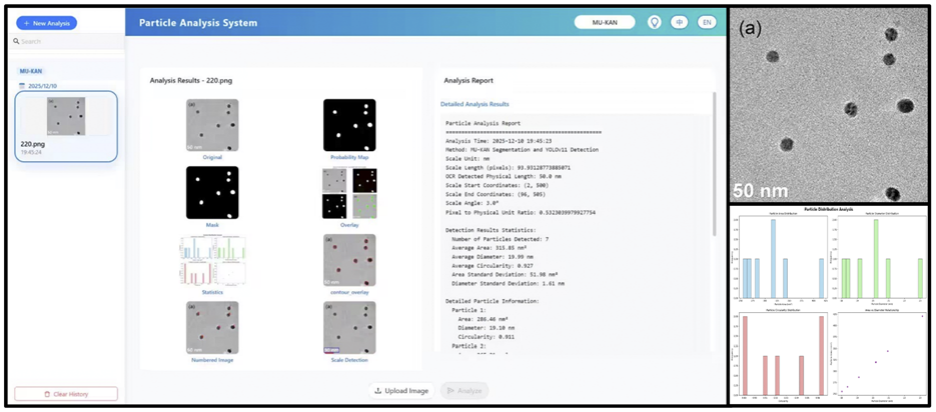
Web interface example.

To demonstrate practical deployment of the proposed framework, a lightweight web interface was developed, as shown in Fig. 5. The interface implements the complete workflow described above and allows users to perform nanoparticle segmentation, scale-bar calibration, and quantitative particle analysis through a unified pipeline. The web interface serves solely as an implementation and visualization platform for the proposed framework and is not considered a methodological contribution.

Additional qualitative examples covering diverse particle morphologies, particle densities, image contrasts, and scale-bar styles are provided in SI (Fig. S1–S10), further demonstrating the applicability of the proposed framework in practical SEM nanoparticle analysis scenarios.

### 3.2 Scale-bar recognition and calibration accuracy

#### 3.2.1 Quantitative comparison of scale-bar recognition strategies

Accurate physical-size calculation requires reliable scale-bar recovery to convert pixel-level statistics into physical units. Three paradigms were evaluated for scale-bar detection and length estimation: a U-Net (ResUNet) semantic segmentation approach, a ViT-based regression approach, and a YOLOv11 bounding-box detection approach. Relative errors are reported in Table 1.

**Table 1:** Scale-bar recognition performance of different models

Methods	Relative error percent (%)
U-Net (ResUNet)	65.3562
Vit	33.9197
YOLOv11	3.8604

As summarized in Table 1, the U-Net (ResUNet) approach exhibits a high relative error of 65.3562%, indicating insufficient robustness under diverse SEM annotation styles and low-quality scale-bar appearances. The Vision Transformer (ViT)-based approach reduces the error to 33.9197% but still shows large deviations in difficult cases.^33^ The YOLOv11 approach achieves a substantially lower relative error of 3.8604%, demonstrating superior reliability for scale-bar localization and pixel-length recovery. These results justify the selection of YOLOv11 as the core detector for scale recovery.

#### 3.2.2 Consistency across scale-length ranges

**Fig. 6:**
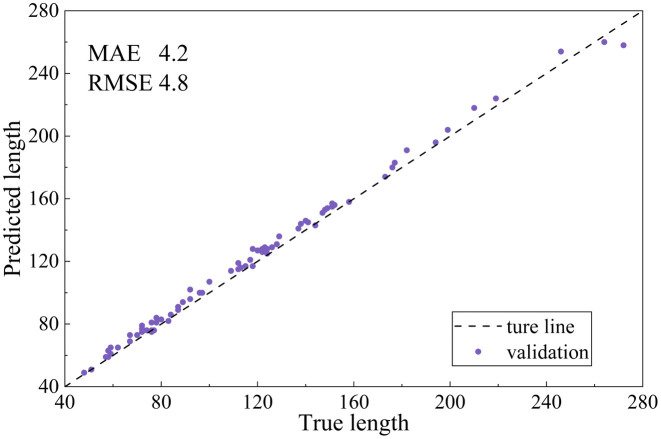
Performance of scale-bar recognition (relative error and correlation).

To evaluate prediction consistency across different ground-truth scale lengths, the predicted *versus* ground-truth scale-bar lengths are shown in Fig. 6. Quantitatively, the YOLOv11-based scale recovery module achieved a mean relative error of 3.8604%, mean absolute error (MAE) of 4.2 pixels, and root mean square error (RMSE) of 4.8 pixels, indicating stable scale-length estimation across the evaluated range. This stability supports reliable pixel-to-physical calibration for practical SEM datasets.

#### 3.2.3 Benchmark summary and ImageJ comparison

Performance was evaluated on NanoSEM-464 and NanoSEM-1707 following the dataset split settings described in the Methods section. Standard pixel-level segmentation metrics were reported, including IoU, F1-score, accuracy, and precision, together with the training loss to reflect optimization stability. In addition to segmentation quality, the practical usability of SEM-based nanoparticle metrology requires reliable pixel-to-physical conversion; therefore, scale-bar recognition and calibration were further evaluated on an in-house annotated scale-bar dataset constructed from NanoSEM-464, consisting of 370 SEM images with manually labeled scale-bar pixel lengths and corresponding physical values. Scale-bar recognition performance was assessed using the relative error of scale-length estimation, and prediction consistency was further examined across a wide range of ground-truth scale values using correlation visualization. Table 1 and Fig. 6 summarize the scale-bar calibration results, which were further validated against measurements from the professional software ImageJ, demonstrating the high accuracy of the proposed measurement framework (see SI Section S6 for detailed comparisons).

### 3.3 Particle-size and morphology statistical analysis

#### 3.3.1 Metrological reliability and descriptor coverage

Based on the predicted particle masks, geometric descriptors including particle area, equivalent circular diameter (ECD), and circularity are automatically calculated. Metrology-oriented validation shows that the proposed workflow achieved the highest Boundary F1-score of 0.9128 together with the lowest mean relative errors for ECD and circularity estimation, reaching 4.3165% and 6.7470%, respectively. These results establish a direct link between boundary fidelity and the reliability of physical-size and particle-shape measurements. The independent comparison with ImageJ reported in SI Section S6 further supports the consistency of the physical-size quantification, while detailed metric definitions and quantitative comparisons are provided in SI Section S10.

Reliable nanoparticle metrology additionally requires accurate pixel-to-physical calibration. As demonstrated in Section 3.2, the scale-bar recognition module achieved a mean relative error of 3.8604% across diverse SEM annotation styles, contrast conditions, and image qualities. The agreement of scale recovery, boundary-level validation, ECD and circularity errors, and ImageJ-based comparison provides complementary evidence that the reported distributions reflect a complete and reproducible metrology chain rather than segmentation quality alone.

Because the benchmark datasets used in this study predominantly contain approximately circular nanoparticles, ECD was adopted as the primary size descriptor for statistical analysis. Nevertheless, the proposed framework is not restricted to ECD-based characterization. To better describe elongated and irregular particles, additional morphology descriptors including major axis length, minor axis length, aspect ratio, Feret diameter, minimum Feret diameter, solidity, and extent were incorporated into the particle analysis module. These descriptors provide complementary information regarding particle elongation, anisotropy, compactness, and deviations from circular morphology. Representative examples and statistical results are provided in SI Section S5.

In addition, incomplete particles located at image boundaries are automatically identified during the particle analysis stage. Particles whose bounding boxes intersect or lie within a predefined distance from the image boundaries are labeled as boundary-truncated particles. These particles are retained in the segmentation visualization results and counted separately, but are excluded from quantitative size and morphology statistics to avoid systematic underestimation caused by partial particle observations. This strategy improves the reliability of the reported particle-size distributions and morphology measurements.

#### 3.3.2 Materials interpretation of particle-size and morphology distributions

As shown in Fig. 4(a), most particles are distributed within an area range of approximately 500–1200 nm^2^, while only a small number exhibit substantially larger projected areas. This concentrated area distribution provides a quantitative basis for assessing particle-population uniformity and identifying oversized outliers, both of which are useful for monitoring synthesis consistency and batch-to-batch variation.

Fig. 4(b) shows that the equivalent circular diameter is primarily distributed between 25 nm and 35 nm, indicating a relatively narrow particle-size distribution. From a materials-characterization perspective, this distribution indicates comparatively uniform particle growth and provides a practical measure of synthesis control. Such uniformity can be important in size-sensitive material systems because surface-to-volume ratio, surface activity, and optical response may vary strongly with particle diameter. The present measurements do not by themselves establish functional performance, but they provide the quantitative structural basis needed for subsequent structure–property correlation.

The circularity distribution shown in Fig. 4(c) is concentrated around 0.8–1.0, indicating that most particles exhibit regular and approximately equiaxed morphologies. Only a small fraction show lower circularity values, suggesting that severe aggregation or highly elongated structures are relatively uncommon in this representative sample. Circularity therefore complements particle diameter by distinguishing changes in shape that may arise from anisotropic growth, aggregation, or variations in synthesis conditions, even when nominal particle sizes are similar.

Fig. 4(d) illustrates the relationship between particle area and equivalent circular diameter. As expected from geometric principles, particle area increases nonlinearly with diameter. Agreement with the theoretical area–diameter relationship provides an internal consistency check for the measurement workflow and supports the reliability of the extracted descriptors for high-throughput sample comparison and quality-control screening.

More importantly, Fig. 4 demonstrates the final metrology output of the proposed framework.^34^ Beyond semantic segmentation, the framework automatically converts segmented particle regions into quantitative size and morphology statistics, enabling direct nanoparticle characterization from SEM images.

### 3.4 Segmentation benchmark comparison on NanoSEM-464 and NanoSEM-1707

The following segmentation benchmarks, loss-function experiments, and boundary visualizations are presented as technical validation of the physical metrology results above. Their purpose is to verify that particle-size and morphology statistics are supported by stable masks and reliable boundaries across varied SEM conditions, rather than to position architecture comparison as the primary endpoint of the study.

#### 3.4.1 Quantitative results on NanoSEM-464

A broad set of semantic segmentation baselines was selected to cover mainstream design paradigms, including lightweight CNN models,^35^ classical U-Net variants and pyramid/multi-scale feature fusion networks,^36^ as well as atrous convolution-based methods, transformer-enhanced hybrids, and KAN-based baselines.^37^ This selection enables comparison across different inductive biases (local convolution, multi-scale aggregation, global context modeling, and nonlinear functional representation).

As summarized in Table 2, the proposed model achieves the best overall performance on NanoSEM-464, reaching an IoU of 0.9296, an F1-score of 0.9630, and an accuracy of 0.9712. A clear margin is observed relative to typical CNN baselines such as DeepLabv3+ (IoU = 0.8031). Compared with the strongest competing method U-KAN (IoU = 0.9033), an absolute IoU gain of 0.0263 is obtained. The results support the effectiveness of multi-scale encoding and the nonlinear bottleneck design for handling blurred boundaries, background noise, and local grayscale fluctuations in SEM images.

**Table 2:** Performance comparison on the NanoSEM-464 validation set

Methods	IoU	Loss	F1	Accuracy	Precision
BiSeNetV2 (ref. 38)	0.6106	1.2255	0.7051	0.8661	0.7050
U-Net (Dense^)^^39^	0.6251	0.5920	0.6833	0.8531	0.6563
DCSAU-Net^40^	0.6269	0.5675	0.7153	0.8722	0.7212
U-Net (ResUNet)^41^	0.6810	0.5114	0.7532	0.8923	0.7372
U-Net (VGG)^42^	0.7115	0.6019	0.6940	0.9041	0.7631
FPN^43^	0.7884	0.4483	0.8684	0.9351	0.8693
deeplabv3plus^44^	0.8031	0.3987	0.8784	0.9401	0.8841
TransUNet^45^	0.8055	0.3961	0.8472	0.9402	0.8652
U-KAN^46^	0.9033	0.1431	0.9476	0.9626	0.9480
MU-KAN (this work)	0.9296	0.1031	0.9630	0.9712	0.9604

#### 3.4.2 Quantitative results on NanoSEM-1707

To further assess robustness on a larger and more diverse dataset, all methods were evaluated on NanoSEM-1707. The results are summarized in Table 3. The proposed model again achieves the best performance with IoU = 0.8243 and F1 = 0.9029. While absolute scores decrease relative to NanoSEM-464, reflecting the increased difficulty and distribution shift in a larger corpus, the performance advantage remains consistent. In particular, an IoU improvement of 0.0251 is achieved over the second-best method Dense U-Net (IoU = 0.7992), indicating improved generalization across variations in particle size, morphology, and contrast.

**Table 3:** Performance comparison on the NanoSEM-1707 validation set

Methods	IoU	Loss	F1	Accuracy	Precision
BiSeNetV2 (ref. 38)	0.4906	1.1247	0.5791	0.9605	0.6946
U-Net (Dense)^39^	0.7992	0.5015	0.8872	0.9857	0.8797
DCSAU-Net^40^	0.7546	0.2259	0.8458	0.9823	0.9118
V-Net (ResUNet)^41^	0.7252	0.2580	0.8147	0.9807	0.8456
U-Net (VGG)^42^	0.7688	0.4598	0.8769	0.9830	0.8330
FPN^43^	0.6738	0.3211	0.7722	0.9768	0.8237
deeplabv3plus^44^	0.7204	0.2694	0.7969	0.9804	0.8526
TransUNet^45^	0.7835	0.2062	0.8767	0.9853	0.8971
U-KAN^46^	0.7922	0.1971	0.8821	0.9851	0.8695
MU-KAN (this work)	0.8243	0.1894	0.9029	0.9879	0.8914

### 3.5 Loss function and robustness analysis

#### 3.5.1 Training convergence and robustness

**Fig. 7:**
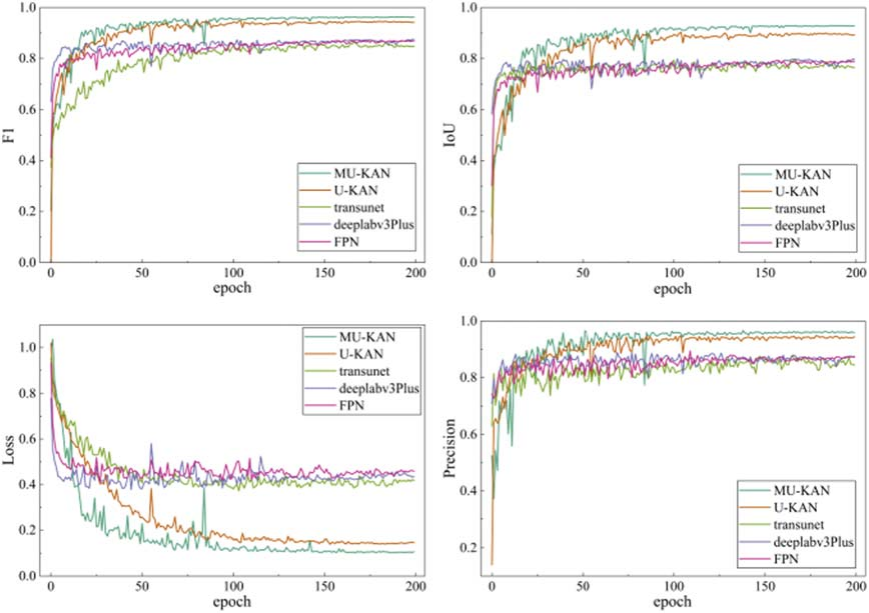
Training curves (F1, IoU, loss, and precision) for MU-KAN and representative baselines on NanoSEM-464.

Training dynamics on NanoSEM-464 are shown in Fig. 7. The loss decreases steadily and gradually stabilizes, while IoU and F1-score increase synchronously and remain stable in later epochs. Compared with representative baselines (*e.g.*, U-KAN, TransUNet, DeepLabv3+, and Feature Pyramid Network (FPN)), the proposed model shows smoother convergence and more stable validation trajectories, indicating improved optimization behavior under complex SEM distributions that involve low contrast, noise, and particle agglomeration.

#### 3.5.2 Loss-function comparison

Foreground–background imbalance is commonly encountered in nanoparticle segmentation tasks. Therefore, the influence of the loss function on segmentation performance was further investigated. In addition to the adopted BCE loss, focal loss was evaluated on both NanoSEM-464 and NanoSEM-1707 under identical training settings, including the same dataset split, training configuration, data augmentation strategy, and random seed. The results obtained on NanoSEM-464 are summarized in Table 4, while the corresponding results on NanoSEM-1707 are provided in SI Table S2.

**Table 4:** Benchmark results of different segmentation architectures on NanoSEM-464 under focal-loss optimization

Methods	IoU	Loss	F1	Accuracy	Precision
BiSeNetV2 (ref. 38)	0.4411	0.0671	0.6064	0.8261	0.7633
U-Net (Dense^)^^39^	0.6757	0.0164	0.8011	0.8938	0.7768
DCSAU-Net^40^	0.6656	0.0154	0.7953	0.8927	0.7839
U-Net (ResUNet)^41^	0.6725	0.0505	0.8003	0.8960	0.7910
U-Net (VGG)^42^	0.6943	0.0306	0.8147	0.9088	0.8352
FPN^43^	0.7643	0.0281	0.8626	0.9264	0.8823
deeplabv3plus^44^	0.7852	0.0246	0.8769	0.9349	0.8983
TransUNet^45^	0.7788	0.0199	0.8722	0.9329	0.8943
U-KAN^46^	0.9123	0.0085	0.9506	0.9665	0.9685
MU-KAN (this work)	0.9188	0.0062	0.9545	0.9682	0.9403

As shown in Table 4, focal loss did not provide consistent performance improvements across the evaluated architectures. For most models, segmentation performance either remained comparable or decreased after replacing BCE with focal loss. For the proposed MU-KAN framework, the IoU decreased from 0.9296 to 0.9188 and the F1-score decreased from 0.9630 to 0.9545. Similar observations were obtained on NanoSEM-1707 (SI Table S2), where the IoU decreased from 0.8243 to 0.8197 and the F1-score decreased from 0.9029 to 0.8932.

These observations suggest that the effectiveness of focal loss is highly dependent on the characteristics of the segmentation task and does not necessarily translate into improved performance for SEM nanoparticle images. In this study, focal loss failed to provide consistent gains in IoU and F1-score across the two benchmark datasets. One possible reason is that SEM nanoparticle images contain numerous ambiguous pixels located near weak-contrast boundaries, noisy regions, and partially agglomerated particles. By assigning greater weights to these hard samples, focal loss may overemphasize local uncertainties during optimization and reduce the contribution of well-classified particle regions. Consequently, the overall segmentation accuracy and mask overlap are not consistently improved. In contrast, BCE loss provides more stable optimization behavior and achieves better overall performance for the proposed MU-KAN framework across both benchmark datasets. Therefore, BCE loss was retained as the default optimization objective throughout this work.

### 3.6 Qualitative boundary consistency analysis

**Fig. 8:**
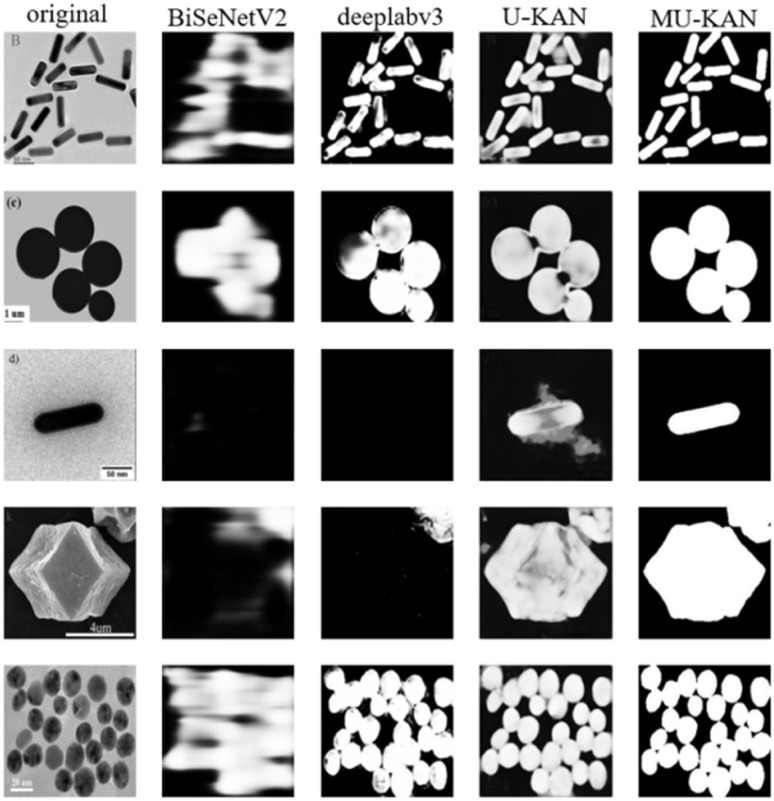
Representative qualitative comparison of segmentation results.

Quantitative metrics do not fully characterize segmentation failure modes in challenging SEM cases, such as weak contrast, dense agglomeration, and small-particle populations.^47^ Representative visual comparisons are presented in Fig. 8.

As shown in Fig. 8, lightweight baselines (*e.g.*, BiSeNetV2) tend to produce fragmented masks or blurred boundaries and often miss fine structures under noisy backgrounds. DeepLabv^3+^ typically captures coarse foreground regions but frequently yields incomplete boundary delineation and misclassification in dense clusters. In contrast, the proposed model produces more continuous and coherent particle boundaries and preserves more small-particle contours than baseline methods, consistent with the quantitative gains in IoU and F1-score. Such boundary consistency is critical for downstream particle-size estimation and morphology descriptor extraction.

### 3.7 Preliminary TEM extension

To preliminarily evaluate the extensibility of the proposed framework beyond SEM images, MU-KAN was further applied to six representative transmission electron microscopy (TEM) images collected from publicly available sources. Representative results are provided in SI (Fig. S11–S16).

Although TEM images differ substantially from SEM images in terms of contrast formation mechanisms, background appearance, and particle-boundary visibility, the proposed framework was able to generate reasonable particle segmentation and preliminary morphology analysis results in most cases. The segmentation masks generally preserved particle morphology and enabled subsequent particle-size analysis, suggesting that the learned representations possess a certain degree of cross-domain applicability.

Nevertheless, the current TEM evaluation remains preliminary. Performance degradation was observed in several challenging cases, particularly for particles exhibiting weak boundaries, dense aggregation, low contrast, or partial occlusion by scale-bar annotations. These imaging characteristics may reduce boundary discrimination capability and subsequently affect morphology measurement accuracy. Representative examples are shown in SI (Fig. S11–S16).

Overall, the preliminary TEM experiments demonstrate the potential applicability of the proposed framework beyond SEM imaging while also highlighting opportunities for further improvement. Future work will involve larger-scale validation on dedicated TEM datasets and the development of microscopy-specific adaptation strategies to further enhance robustness across different electron microscopy modalities.

Additional cross-domain experiments are provided in the SI to assess architectural robustness.

### 3.8 Practical applications and deployment scenarios

The proposed SEM-based nanoparticle metrology framework is designed for practical deployment in a wide range of materials science and engineering applications. In catalyst characterization, the automated extraction of particle size distributions and morphological descriptors enables quantitative analysis of morphology–activity relationships, providing a basis for catalyst design, optimization, and performance evaluation. In battery materials research, statistically reliable measurements of nanoparticle size and shape contribute to understanding electrode microstructure evolution and its impact on electrochemical properties and degradation behavior.

In nanomaterial synthesis, the framework can be integrated into experimental workflows to provide rapid and consistent feedback on particle morphology, thereby facilitating optimization of synthesis parameters and improving reproducibility across different experimental batches. In addition, for industrial quality control of nanomaterials, the scale-calibrated measurement pipeline ensures accurate conversion from pixel-level measurements to physical dimensions, enabling standardized and operator-independent characterization under varying SEM imaging conditions.

Moreover, due to its fully automated and high-throughput design, the proposed framework is suitable for large-scale structure–property studies where statistically robust characterization of nanoparticle populations is required across diverse datasets. This improves both efficiency and reproducibility in SEM-based materials characterization workflows, particularly in scenarios involving large image datasets and cross-domain experimental conditions.

## 4 Conclusion

Quantitative links between nanoparticle morphology and materials performance require SEM measurements that are accurate, reproducible, and scalable. In this work, we developed an automated SEM-based nanoparticle metrology workflow that integrates MU-KAN particle segmentation, YOLOv11-based scale-bar localization, OCR-assisted scale annotation parsing, and pixel-to-physical conversion. Rather than treating segmentation as an endpoint, the framework converts raw SEM micrographs into physically meaningful particle descriptors, including particle area, equivalent circular diameter, circularity, and extended morphology metrics, thereby supporting high-throughput nanoparticle characterization.

The proposed framework achieved robust segmentation performance on two public SEM nanoparticle benchmarks, with IoU values of 0.9296 and 0.8243 and F1-scores of 0.9630 and 0.9029 on NanoSEM-464 and NanoSEM-1707, respectively. The scale-bar recovery module achieved a mean relative error of 3.8604%, enabling reliable calibration from pixel units to physical dimensions. Importantly, the improved boundary quality translated into downstream metrology accuracy, as reflected by the highest Boundary F1-score of 0.9128 and the lowest relative errors for equivalent circular diameter and circularity estimation. These results demonstrate that the integrated workflow can provide consistent particle-size and morphology statistics rather than segmentation masks alone.

Despite these advances, several challenges remain for highly agglomerated particles,^48^ weak inter-particle boundaries, strongly anisotropic morphologies, and severely degraded or unconventional scale annotations. Future work will focus on instance-aware particle separation, uncertainty-aware calibration, morphology-specific descriptors, and broader validation across TEM and other electron microscopy modalities. Overall, this framework provides a practical and reproducible route for automated nanoparticle metrology, with potential utility in catalyst characterization, battery-material analysis, nanomaterial synthesis optimization, and quality control.

## Author contributions

X. Y. H.: contributed to investigation, formal analysis, and writing – original draft. Y. Z. Y.: contributed to investigation, formal analysis, and writing – original draft. J. H. H.: participated in investigation and formal analysis. W. X. Y.: performed formal analysis. L. F.: conducted formal analysis. J. L.: was responsible for conceptualization, funding acquisition, writing – original draft, and writing – review and editing. D. D. L.: provided supervision.

## Conflicts of interest

There are no conflicts to declare.

## Supplementary Material

RA-OLF-D6RA02763F-s001

## Data Availability

Data supporting this study, including SEM datasets, annotations, scale-bar benchmarks, morphology measurements, and code, are available at https://github.com/hxy434/MU-KAN. Supplementary information (SI): additional SEM image examples, experimental details, segmentation results, quantitative comparisons, correlation analyses, and validation results. See DOI: https://doi.org/10.1039/d6ra02763f.

## References

[cit1] Yan X., Zhou Y., Wang S. (2025). Nano-High Entropy Materials in Electrocatalysis. Adv. Funct. Mater..

[cit2] Dalbanjan N. P., Korgaonkar K., Eelager M. P., Gonal B. N., Kadapure A. J., Arakera S. B., Kumar S.K P. (2025). In-silico strategies in nano-drug design: Bridging nanomaterials and pharmacological applications. Nano TransMed.

[cit3] Liu Y., Bose S., Fan W. (2018). Effect of size and shape on electronic and optical properties of CdSe quantum dots. Optik.

[cit4] Ma Z., Yin Y., Yin Y., Liu Z., Yin X., Han Y., Hei J., Wang N., Zhang Y. (2025). Pseudo-Oxide Metal Carbodiimides: Potential Burn Rate Modifier for the Decomposition of Ammonium Perchlorate. Propellants, Explos., Pyrotech..

[cit5] Ali A., Zhang N., Santos R. M. (2023). Mineral Characterization Using Scanning Electron Microscopy (SEM): A Review of the Fundamentals, Advancements, and Research Directions. Appl. Sci..

[cit6] Kim H., Han J., Han T. Y.-J. (2020). Machine vision-driven automatic recognition of particle size and morphology in SEM images. Nanoscale.

[cit7] Bals J., Epple M. (2023). Deep learning for automated size and shape analysis of nanoparticles in scanning electron microscopy. RSC Adv..

[cit8] Yang H., Ahuja N. (2014). Automatic segmentation of granular objects in images: Combining local density clustering and gradient-barrier watershed. Pattern Recognit..

[cit9] Ma W., Kautz E. J., Baskaran A., Chowdhury A., Joshi V., Yener B., Lewis D. J. (2021). Image-driven discriminative and generative machine learning algorithms for establishing microstructure–processing relationships. JOM.

[cit10] Zhong Y., Liu Y., Liu K., Zhan T., Liu S., Liang Y., Hu Y., Li M., Lei G., Zhou S., Liu J. (2025). WC electron microscopy image segmentation based on improved watershed and Hu-moment edge matching algorithms. Comput. Mater. Sci..

[cit11] López Gutiérrez J. D., Abundez Barrera I. M., Torres Gómez N. (2022). Nanoparticle Detection on SEM Images Using a Neural Network and Semi-Synthetic Training Data. Nanomaterials.

[cit12] Ede J. M. (2021). Deep learning in electron microscopy, Mach. Learn. Sci. Technol..

[cit13] Ma C., Mi J., Gao W., Tao S. (2024). SSGAN: A Semantic Similarity-Based GAN for Small-Sample Image Augmentation. Neural Process. Lett..

[cit14] Kwong Q. B., Kon Y. T., Rusik W. R. W., Shabudin M. N. A., Rahman S. S. A., Kulaveerasingam H., Appleton D. R. (2024). Enhancing oil palm segmentation model with GAN-based augmentation. J. Big Data.

[cit15] Kugelman J., Alonso-Caneiro D., Read S. A., Vincent S. J., Collins M. J. (2024). Enhancing OCT patch-based segmentation with improved GAN data augmentation and semi-supervised learning. Neural Comput. Appl..

[cit16] Göçeri E. (2024). GAN based augmentation using a hybrid loss function for dermoscopy images. Artif. Intell. Rev..

[cit17] Rettenberger L., Szymanski N. J., Zeng Y., Schuetzke J., Wang S., Ceder G., Reischl M. (2024). Uncertainty-aware particle segmentation for electron microscopy at varied length scales. npj Comput. Mater..

[cit18] Kim T., Yeo B. C. (2023). Recovering Microscopic Images in Material Science Documents by Image Inpainting. Appl. Sci..

[cit19] Gonzalez R. C., Woods R. E., Masters B. R. (2009). Digital Image Processing. J. Biomed. Opt..

[cit20] Liu Z. , Wang Y., Vaidya S., Ruehle F., Halverson J., Soljačić M., Hou T. Y. and KAN T. M., Kolmogorov–Arnold networks, in. International Conference on Learning Representations (ICLR), Machine Learning, 2025

[cit21] Yildirim B., Cole J. M. (2021). Bayesian Particle Instance Segmentation for Electron Microscopy Image Quantification. J. Chem. Inf. Model..

[cit22] Zahedi-Nasab R. , The Particle Masks for Segmentation, Kaggle, 2022, https://www.kaggle.com/datasets/roxanazahedi/particle-array-2

[cit23] ISO 13322-1 , Particle Size Analysis — Image Analysis Methods — Part 1: Static Image Analysis Methods, International Organization for Standardization, Geneva, 2014

[cit24] Moradpur-Tari E., Vlassov S., Oras S., Ernits M., Damerchi E., Polyakov B., Kyritsakis A., Zadin V. (2024). Nano1D: An accurate computer vision software for analysis and segmentation of low-dimensional nanostructures. Ultramicroscopy.

[cit25] Lamprecht M. R., Sabatini D. M., Carpenter A. E. (2007). CellProfiler™: Free, Versatile Software for Automated Biological Image Analysis. BioTechniques.

[cit26] Schneider C. A., Rasband W. S., Eliceiri K. W. (2012). NIH Image to ImageJ: 25 years of image analysis. Nat. Methods.

[cit27] Muñoz-Rodenas J., García-Sevilla F., Miguel-Eguía V., Coello-Sobrino J., Martínez-Martínez A. (2024). A Deep Learning Approach to Semantic Segmentation of Steel Microstructures. Appl. Sci..

[cit28] Baskaran A., Kautz E. J., Chowdhary A., Ma W., Yener B., Lewis D. J. (2021). Adoption of Image-Driven Machine Learning for Microstructure Characterization and Materials Design: A Perspective. JOM.

[cit29] Xu Y., Xu D., Yu N., Liang B., Yang Z., Asif M. S., Yan R., Liu M. (2023). Machine Learning Enhanced Optical Microscopy for the Rapid Morphology Characterization of Silver Nanoparticles. ACS Appl. Mater. Interfaces.

[cit30] LeCun Y., Bengio Y., Hinton G. (2015). Deep learning. Nature.

[cit31] Zahedi R., Bagheri H., Ghasemian F., Ghazvini M., Ziaei S. Y. (2025). Nano-particles size measurement based on semantic segmentation via convolution neural network. Measurement.

[cit32] Sun Z., Shi J., Wang J., Jiang M., Wang Z., Bai X., Wang X. (2022). A deep learning-based framework for automatic analysis of the nanoparticle morphology in SEM/TEM images. Nanoscale.

[cit33] Dosovitskiy A. , *et al.*, An Image is Worth 16x16 Words: Transformers for Image Recognition at Scale, International Conference on Learning Representations (ICLR), 2021

[cit34] Li C., Liu X., Li W., Wang C., Liu H., Liu Y., Chen Z., Yuan Y. (2025). U-KAN Makes Strong Backbone for Medical Image Segmentation and Generation. Proc. AAAI Conf. Artif. Intell..

[cit35] Saaim K. M., Afridi S. K., Nisar M., Islam S. (2022). In search of best automated model: Explaining nanoparticle TEM image segmentation. Ultramicroscopy.

[cit36] Monchot P., Coquelin L., Guerroudj K., Feltin N., Delvallée A., Crouzier L., Fischer N. (2021). Deep Learning Based Instance Segmentation of Titanium Dioxide Particles in the Form of Agglomerates in Scanning
Electron Microscopy. Nanomaterials.

[cit37] Tao T., Ji H., Liu B. (2025). A deep learning method for nanoparticle size measurement in SEM images. RSC Adv..

[cit38] Yu C., Gao C., Wang J., Yu G., Shen C., Sang N. (2021). BiSeNet V2: Bilateral Network with Guided Aggregation for Real-Time Semantic Segmentation. Int. J. Comput. Vis..

[cit39] Jégou S. , Drozdzal M., Vazquez D., Romero A. and Bengio Y., The one hundred layers Tiramisu: Fully convolutional DenseNets for semantic segmentation, IEEE Conference on Computer Vision and Pattern Recognition Workshops (CVPRW), 2017, 10.1109/CVPRW.2017.156

[cit40] Wang J., Chen C., Ding M., Yu Y., Zha H. (2021). DCSAU-Net: Dense convolutional spatial attention U-Net for medical image segmentation. Comput. Methods Progr. Biomed..

[cit41] Zhang Z., Liu Q., Wang Y. (2018). Road Extraction by Deep Residual U-Net. IEEE Geosci. Remote Sens. Lett..

[cit42] Simonyan K. and Zisserman A., Very deep convolutional networks for large-scale image recognition, International Conference on Learning Representations (ICLR), 2015

[cit43] Cambrin D. , Caputo B. and Masone C., U-kan: Tokenized kolmogorov–arnold networks for medical image segmentation, European Conference on Computer Vision Workshops (ECCV Workshops), 2024

[cit44] Głowska A., Jolimaitre E., Hammoumi A., Moreaud M., Sorbier L., de Faria Barros C., Lefebvre V., Coppens M.-O. (2024). Sem image processing assisted by deep learning to quantify mesoporous γ-alumina spatial heterogeneity and its predicted impact on mass transfer. J. Phys. Chem. C.

[cit45] Chen J. , Lu Y., Yu Q., Luo X., Adeli E., Wang Y., Lu L., Yuille A. L. and Zhou Y., Trans UNet: Transformers make strong encoders for medical image segmentation, in. International Conference on Medical Image Computing and Computer-Assisted Intervention (MICCAI), 2021, 12902, pp. 224–234, 10.1007/978-3-030-87196-3_22

[cit46] Li Y. , Wang Z. and Zhang X., arXiv, 2024, preprint, arXiv:2406.02918, 10.48550/arXiv.2406.02918

[cit47] Taha A. A., Hanbury A. (2015). Metrics for evaluating 3D medical image segmentation: analysis, selection, and tool. BMC Med. Imag..

[cit48] Mill L., Wolff D., Gerrits N., Philipp P., Kling L., Vollnhals F., Ignatenko A., Jaremenko C., Huang Y., De Castro O., Audinot J.-N., Nelissen I., Wirtz T., Maier A., Christiansen S. (2021). Synthetic Image Rendering Solves Annotation Problem in Deep Learning Nanoparticle Segmentation. Small Methods.

