# Supplementary material for: Automated SEM-based nanoparticle metrology for materials characterization via segmentation and robust scale-bar recognition
Source: RSC Adv. 2026 Aug 5;16(39):45081–91. doi: 10.1039/d6ra02763f (PMC13441050; doi:10.1039/d6ra02763f)
Supplement: Supplementary file 1 [file RA-016-D6RA02763F-s001.pdf]

Supplementary Information (SI) for RSC Advances.
This journal is © The Royal Society of Chemistry 2026

Supporting Information

Automated SEM-Based Nanoparticle Metrology for Materials Characterization via Segmentation and Robust Scale-Bar Recognition

Xueyi Huang[a,1], Yuzhong Yin[b,1], Jinghao Hu[b], Wenxiao Yu[c], Liang Fang[a], Jian Liu[a,*], Dongdong Li[b,*]

Content

S1. Additional Quantitative Evaluation on Public Datasets 3
S2. Additional Qualitative Segmentation Results 4
S3. Preliminary Evaluation on TEM Images 14
S4. Additional Loss Function Analysis 20
S5. Extended Morphological Characterization of Irregular Particles 21
S6. Validation of Particle Size Measurement Accuracy 24
S7. Hyperparameter Optimization and Five-Fold Cross-Validation 32
S8. Effect of input resolution on segmentation performance 36
S9. Comparison of OCR Methods for Scale Annotation Recognition 37
S10. Boundary and Metrology Accuracy Analysis 38
References 40

S1. Additional Quantitative Evaluation on Public Datasets

To further validate the generalization capability of the proposed **MU-KAN** model, additional experiments were conducted on three widely used public medical image segmentation datasets: **BUSI**, **GlaS**, and **CVC**. These datasets are commonly adopted benchmarks for evaluating the robustness and transferability of segmentation models across different medical imaging modalities.

The results demonstrate that the proposed MU-KAN achieves consistently competitive or superior performance across all three datasets. In particular, MU-KAN achieves an IoU of **67.65 ± 2.74** and an F1-score of **80.59 ± 2.43** on the BUSI dataset. On the GlaS dataset, the model attains an IoU of **89.90 ± 0.26** and an F1-score of **94.12 ± 0.21**. On the CVC dataset, MU-KAN achieves an IoU of **85.86 ± 0.61** and an F1-score of **92.26 ± 0.25**.

Table S1 Segmentation Performance on BUSI, GlaS and CVC Datasets

Methods	BUSI		GlaS		CVC	
	IoU	F1	IoU	F1	IoU	F1
U-Net	57.22±4.74	71.91±3.54	86.66±0.91	92.79±0.56	83.79±0.77	91.06±0.47
Att-Unet	55.18±3.61	70.22±2.88	87.04±1.76	92.98±0.65	84.52±0.51	91.46±0.25
U-Net++	57.41±4.77	72.11±3.90	86.83±0.91	92.86±0.44	84.61±1.47	91.53±0.88
U-NeXt	59.06±1.03	73.08±1.32	84.51±0.37	91.55±0.23	74.83±0.24	85.36±0.17
Rolling-UNet	61.00±0.64	74.67±1.24	86.42±0.96	92.63±0.62	82.87±1.42	90.48±0.83
U-Mamba	61.81±3.24	75.55±3.01	87.01±0.39	93.02±0.24	84.79±0.58	91.63±0.39
U-KAN	63.38±2.83	76.40±2.90	87.64±0.32	93.37±0.16	85.05±0.53	91.88±0.29
**MU-KAN(Ours)**	**67.65±2.74**	**80.59±2.43**	**89.90±0.26**	**94.12±0.21**	**85.86±0.61**	**92.26±0.25**

The quantitative comparison results are summarized in Table S1. For fair comparison, the reported performance of several baseline models (U-Net, Attention U-Net, U-Net++, U-NeXt, Rolling-UNet, U-Mamba, and U-KAN) on the BUSI, GlaS, and CVC datasets are adopted from the AAAI 2025[1], where all models were evaluated under consistent experimental settings.

These results indicate that the proposed model maintains strong segmentation performance across different datasets and imaging domains, demonstrating its robustness and general applicability beyond nanoparticle SEM images.

S2. Additional Qualitative Segmentation Results

To further illustrate the segmentation performance of the proposed MU-KAN model, we present qualitative segmentation results on ten representative SEM particle images, as shown in **Fig. S1–S10**. In these ten images, A represents the original SEM micrograph, B represents the semantic segmentation map, C represents the scale bar detection result, and D represents the output of the web page designed in this paper for these SEM particle images.These examples provide a visual comparison between the baseline segmentation model and the proposed method.

The ten micrographs (Fig. S1–S10) exhibit substantial diversity in particle morphology, size, and imaging conditions, which pose significant challenges for automated semantic segmentation and scale bar detection:

(1) **Morphological Variability**: Particles range from triangular prisms (Fig. S1) to solid spheres (Fig. S3, S5, S8), hollow nanocavities (Fig. S9), and core–shell structures (Fig. S4, S10). This diversity complicates the definition of consistent segmentation boundaries.

(2) **Size and Scale Range**: The scale bars span from 5 nm (Fig. S5, S6) to 5 μm (Fig. S9), covering three orders of magnitude. Particles range from ~5 nm to several micrometers, requiring the system to handle both high-resolution details and large-field-of-view contexts.

(3) **Contrast and Clarity**: Some images (e.g., Fig. S2, a selected-area electron diffraction pattern) lack distinct particle features, while others (e.g., Fig. S7) show sparse, low-contrast particles against a noisy background, making edge detection difficult.

(4) **Density and Overlap**: Images like Fig. S4 and S10 contain densely packed, overlapping particles, increasing the risk of under-segmentation (merging adjacent objects) or over-segmentation (splitting single particles).

(5) **Scale Bar Inconsistencies**: Scale bars appear in different positions, fonts, and units (nm, μm), with varying line thickness and contrast (e.g., Fig. S9 has a thick white bar, while Fig. S10 has a thin black bar). This requires robust text and symbol recognition.

By successfully processing this diverse set of images, the system demonstrates its broad applicability and robustness across a wide range of nanomaterial characterization scenarios, from high-resolution TEM to large-area SEM.

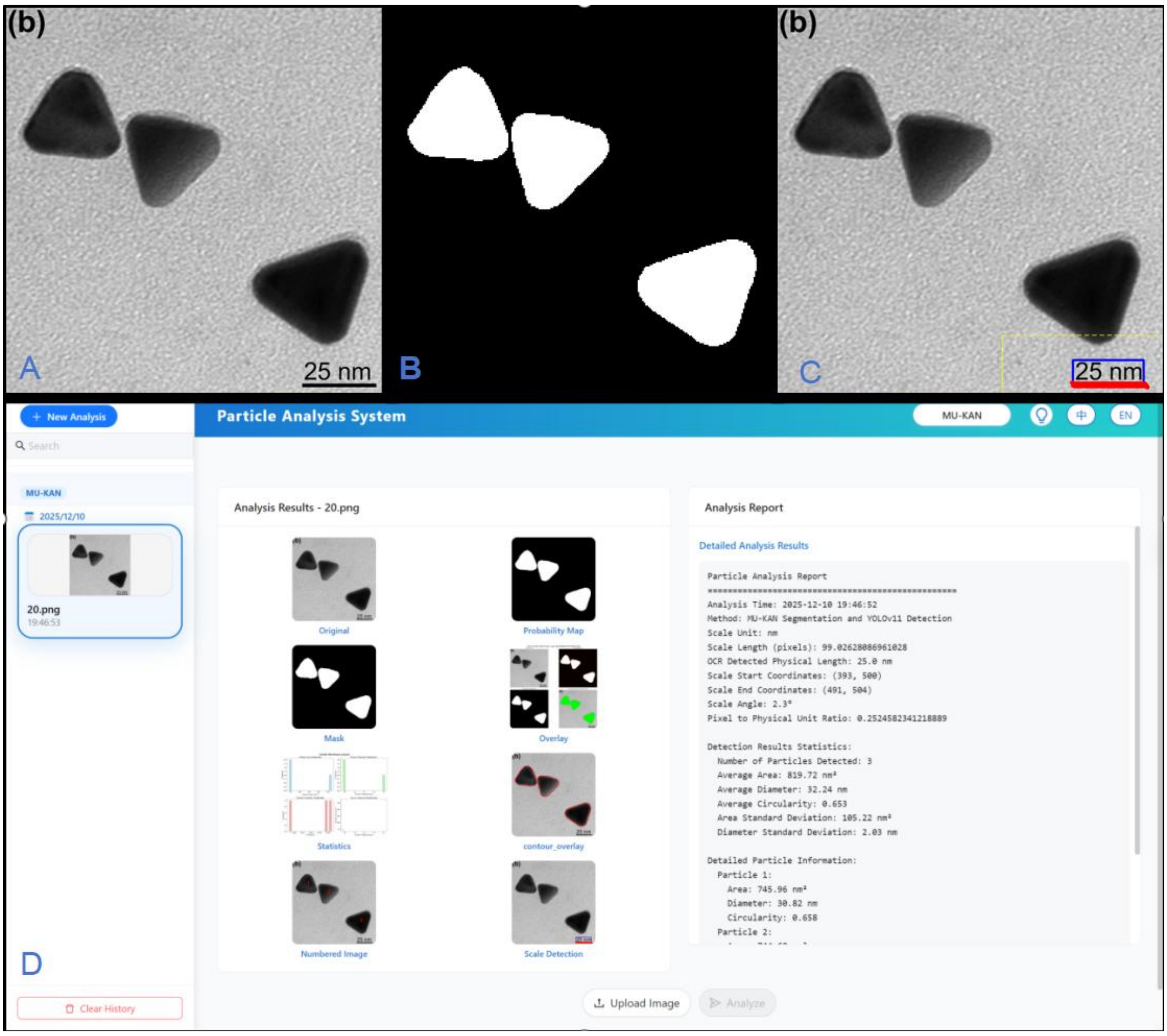

Figure.S1 Example 1.

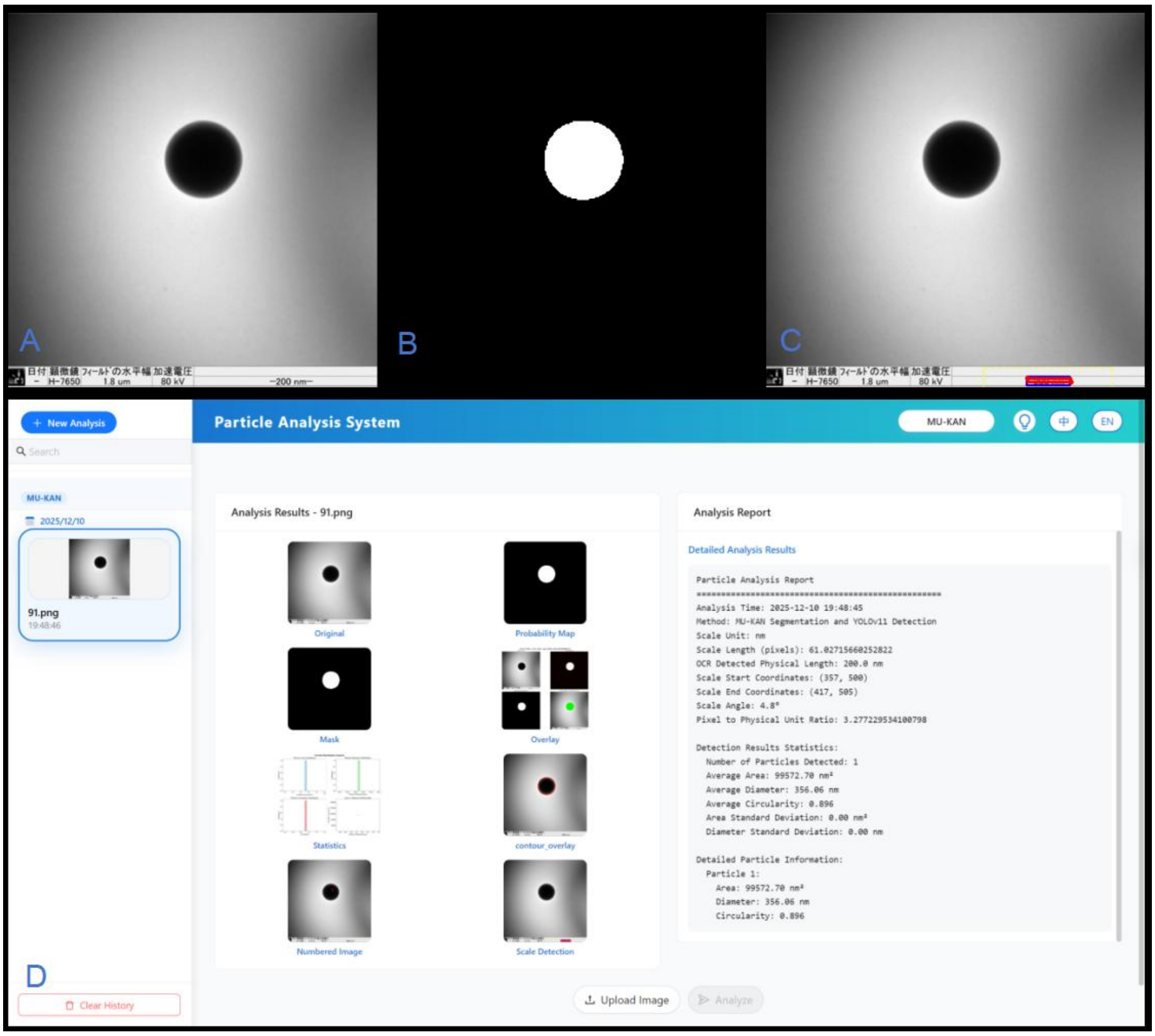

Figure.S2 Example 2.

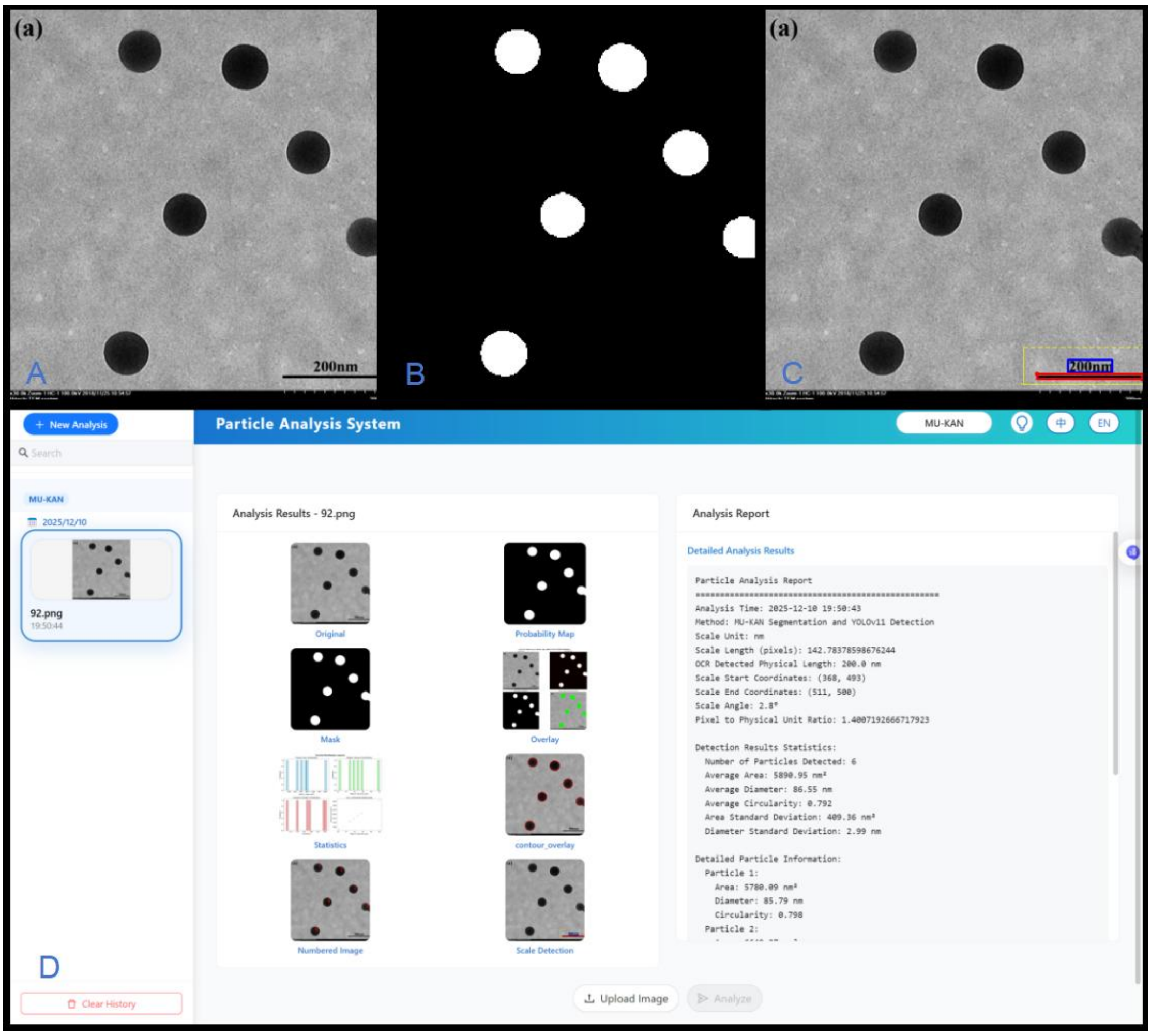

Figure.S3 Example 3.

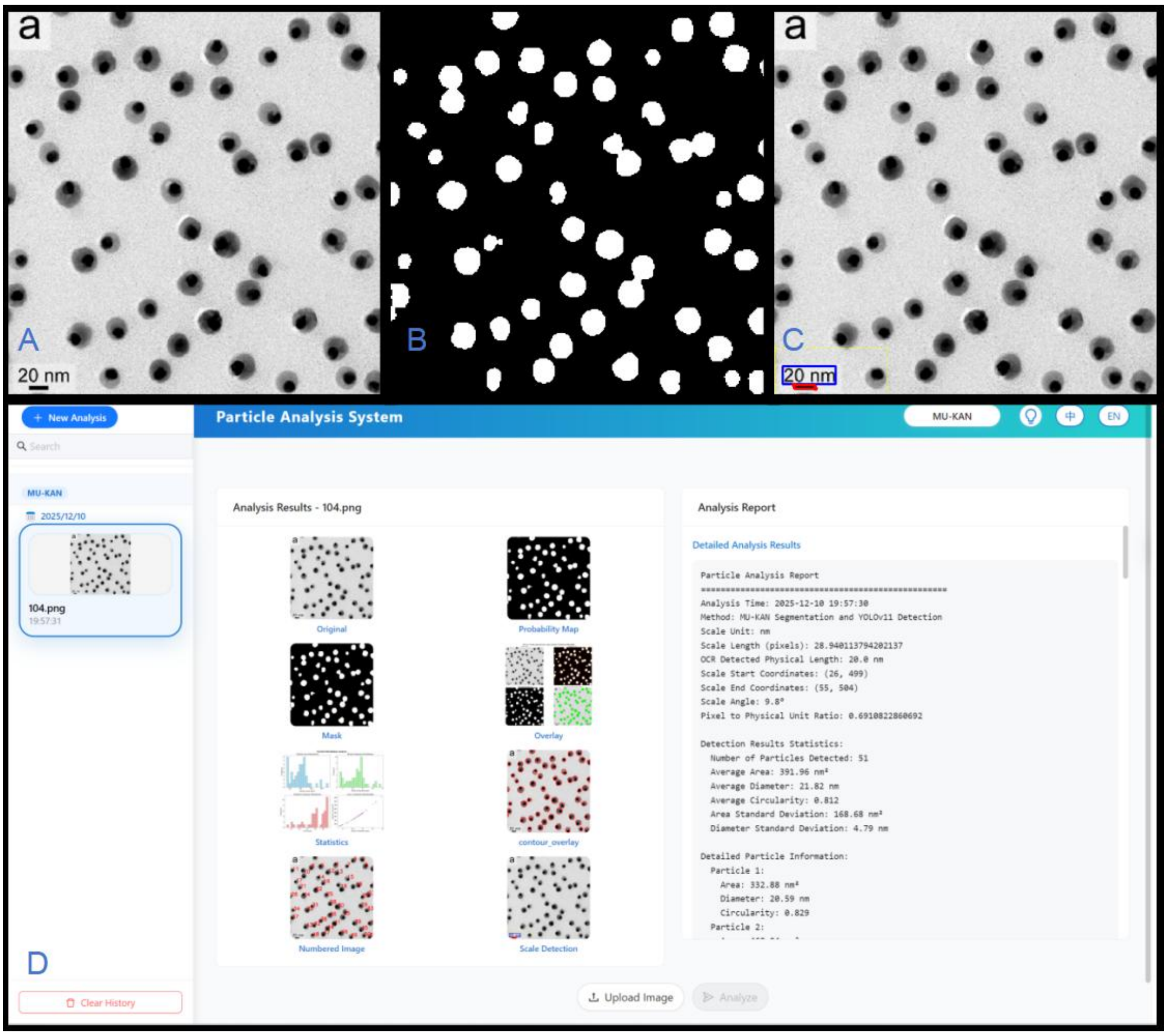

Figure.S4 Example 4.

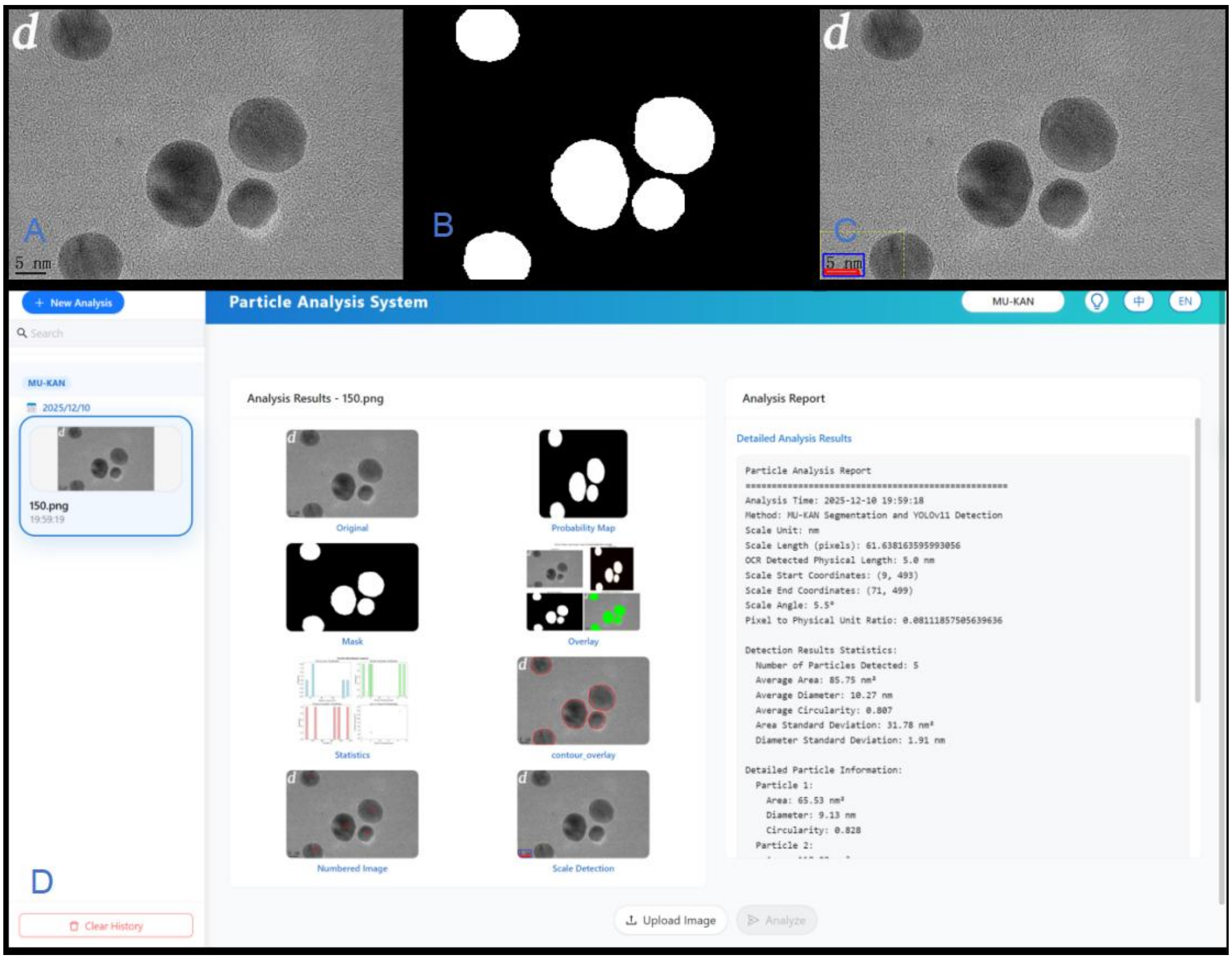

Figure.S5 Example 5.

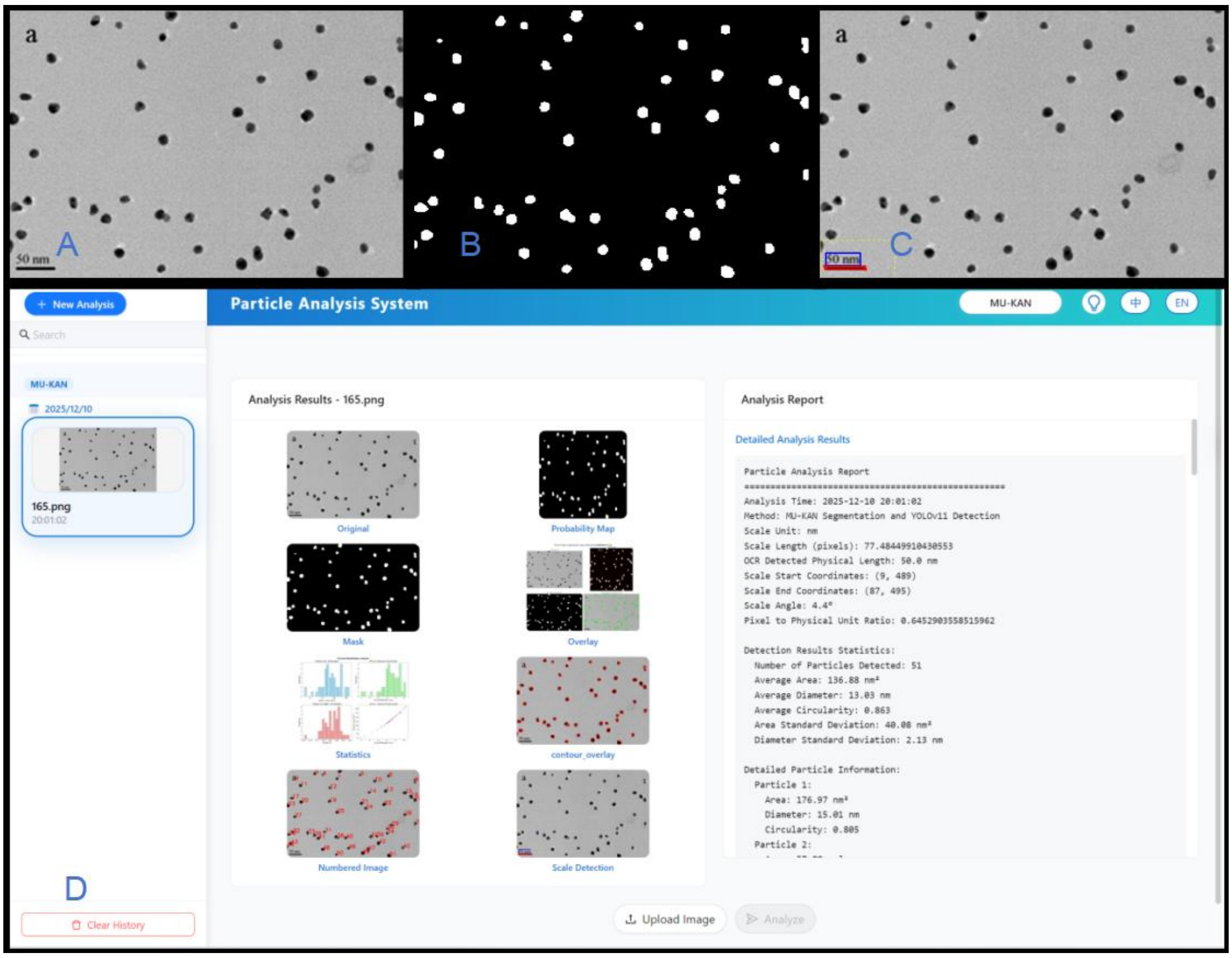

Figure.S6 Example 6.

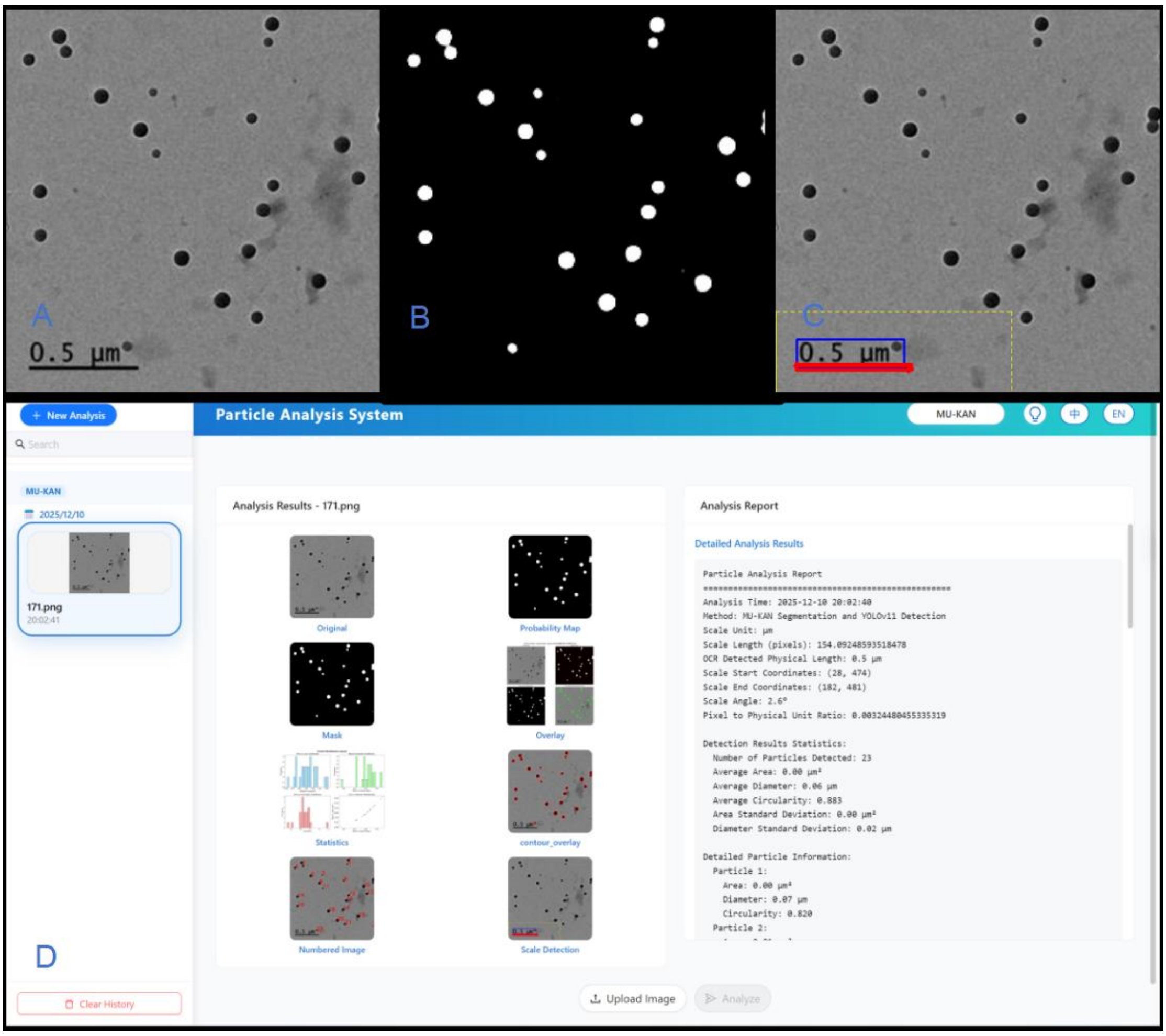

Figure.S7 Example 7.

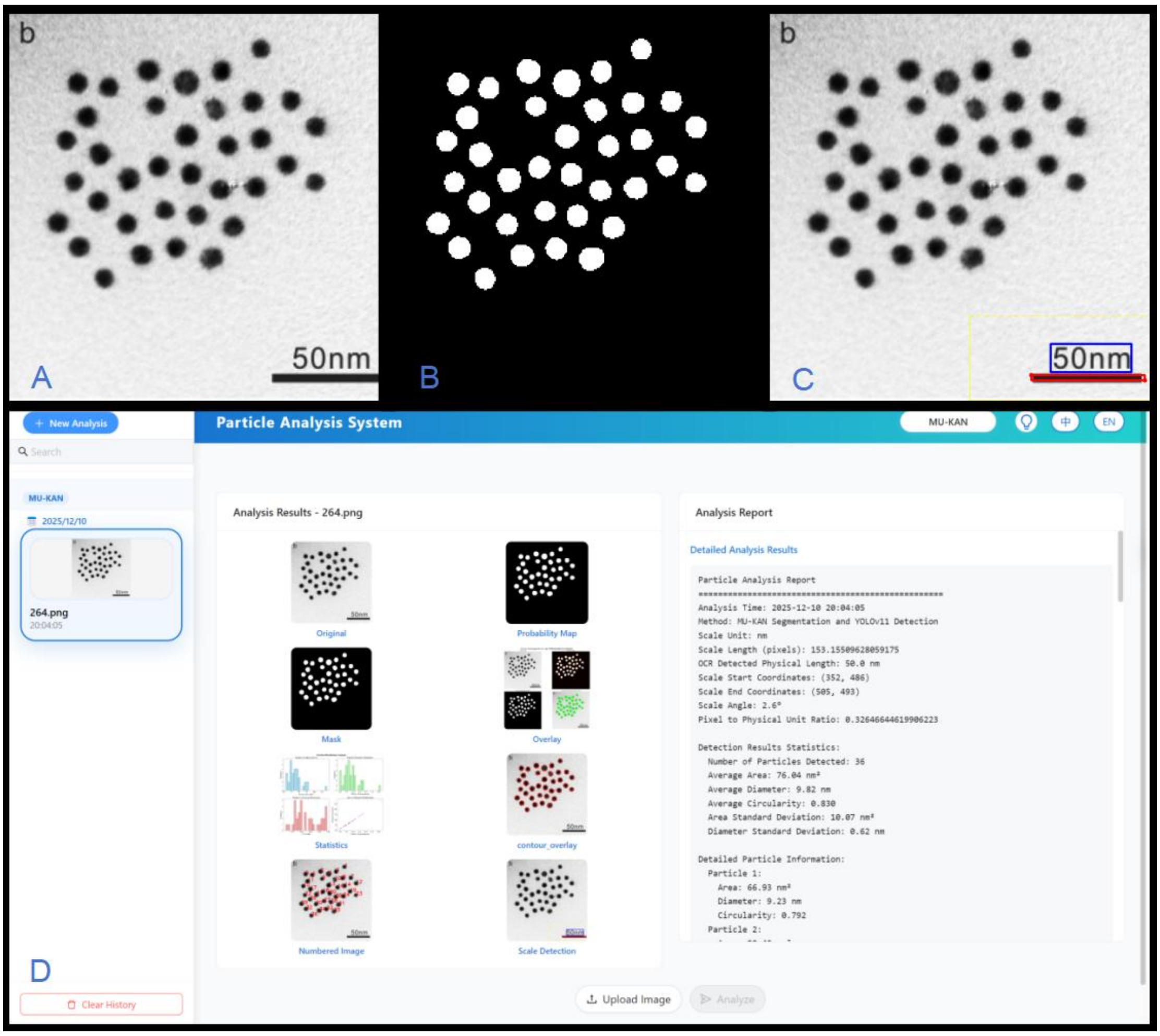

Figure.S8 Example 8.

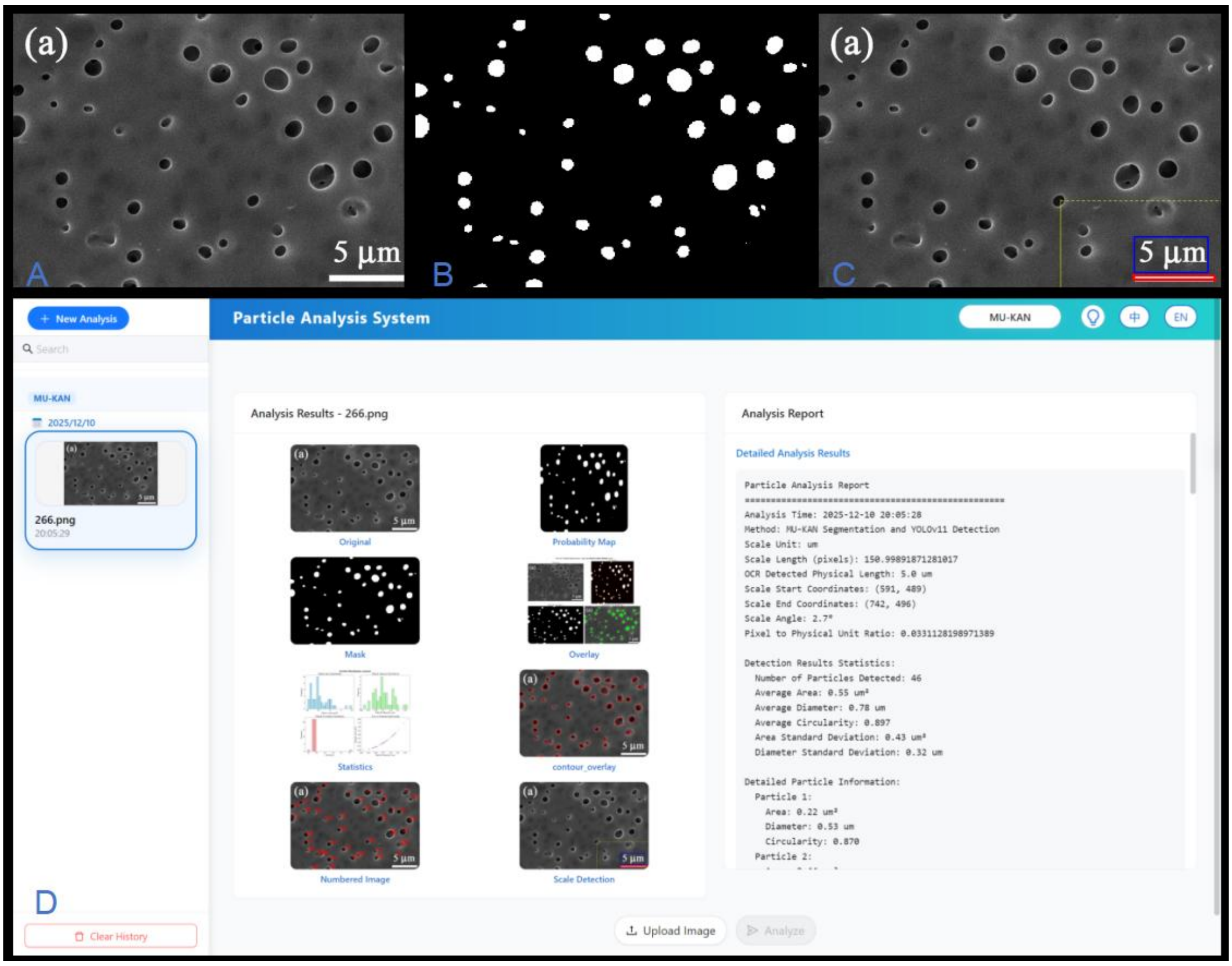

Figure.S9 Example 9.

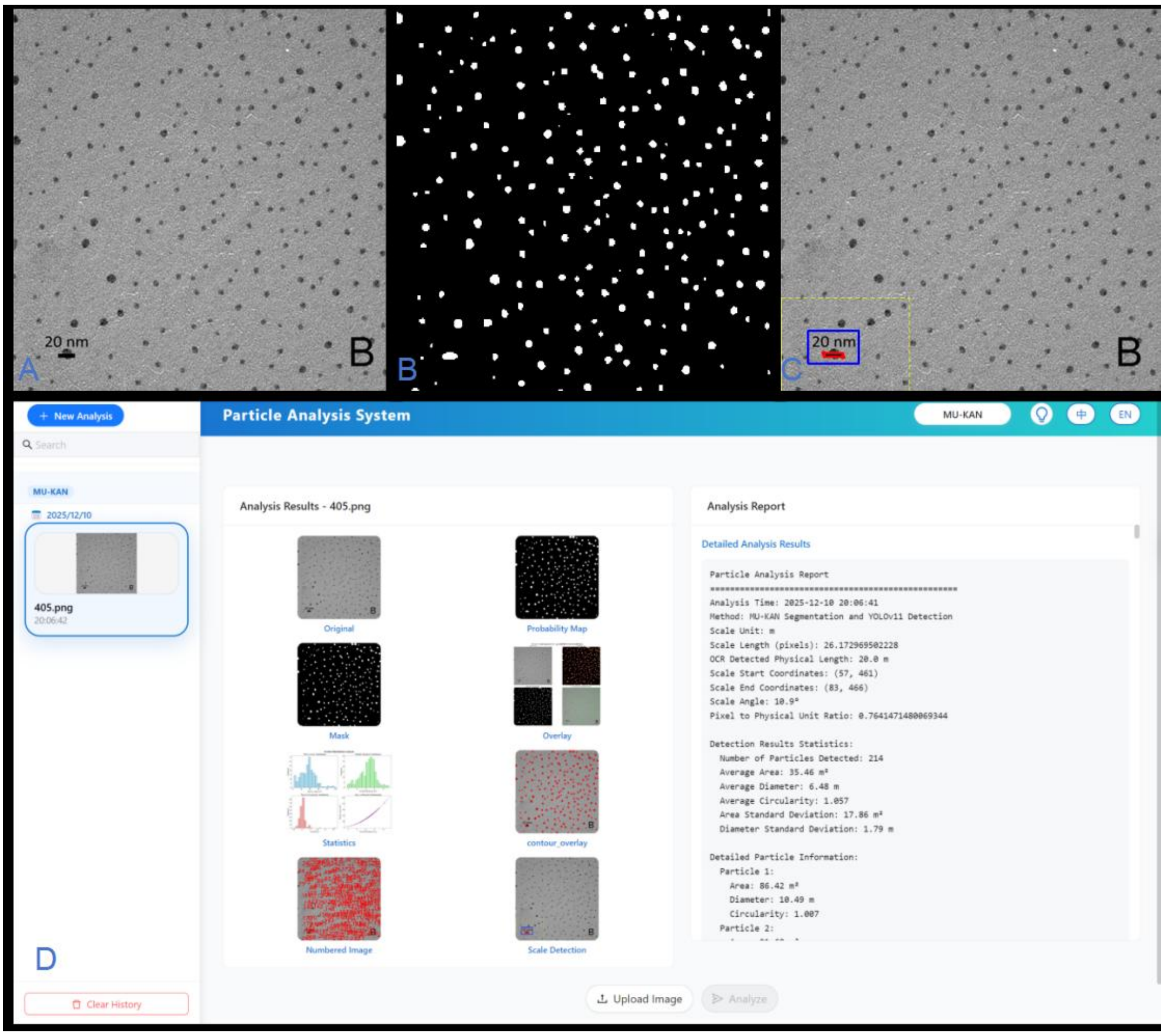

Figure .S10 Example 10.

S3. Preliminary Evaluation on TEM Images

To further investigate the potential applicability of MU-KAN to TEM images, we additionally collected six representative nanoparticle TEM images from publicly available sources, including open-source websites and related publications[2], and performed preliminary qualitative evaluation using the proposed framework.

Compared with SEM images, TEM images often exhibit substantially different contrast characteristics, lower boundary contrast, imaging noise distributions, and particle representations. Despite these differences, MU-KAN was still able to identify nanoparticle regions and generate preliminary segmentation and particle size analysis results in most cases. However, the segmentation performance became less satisfactory in some complex TEM scenarios, particularly for densely aggregated particles, weak inter-particle boundaries, and partially occluded particles.

Several limitations were also observed during the preliminary TEM evaluation. For example, due to the relatively low contrast of TEM images, some particles partially covered by the scale bar were missed during segmentation (e.g., Fig. S14). In addition, particles with extremely close contact or weak boundaries were occasionally identified as a single connected particle (e.g., Fig. S16).

Since the current framework was primarily developed and optimized for SEM nanoparticle analysis, the present TEM evaluation remains preliminary, and further dedicated optimization and validation on large-scale TEM datasets are still required.

In Fig. S11–S16, A represents the original TEM image and B represents the semantic segmentation mask generated by MU-KAN. Since no scale bars are present in Fig. S11–S13, S15, and S16, panel C shows the overlay visualization of the segmentation mask and the original image. In Fig. S14, where a scale bar is present, panel C represents the scale-bar detection result. Panel D represents the output of the web interface designed in this work.

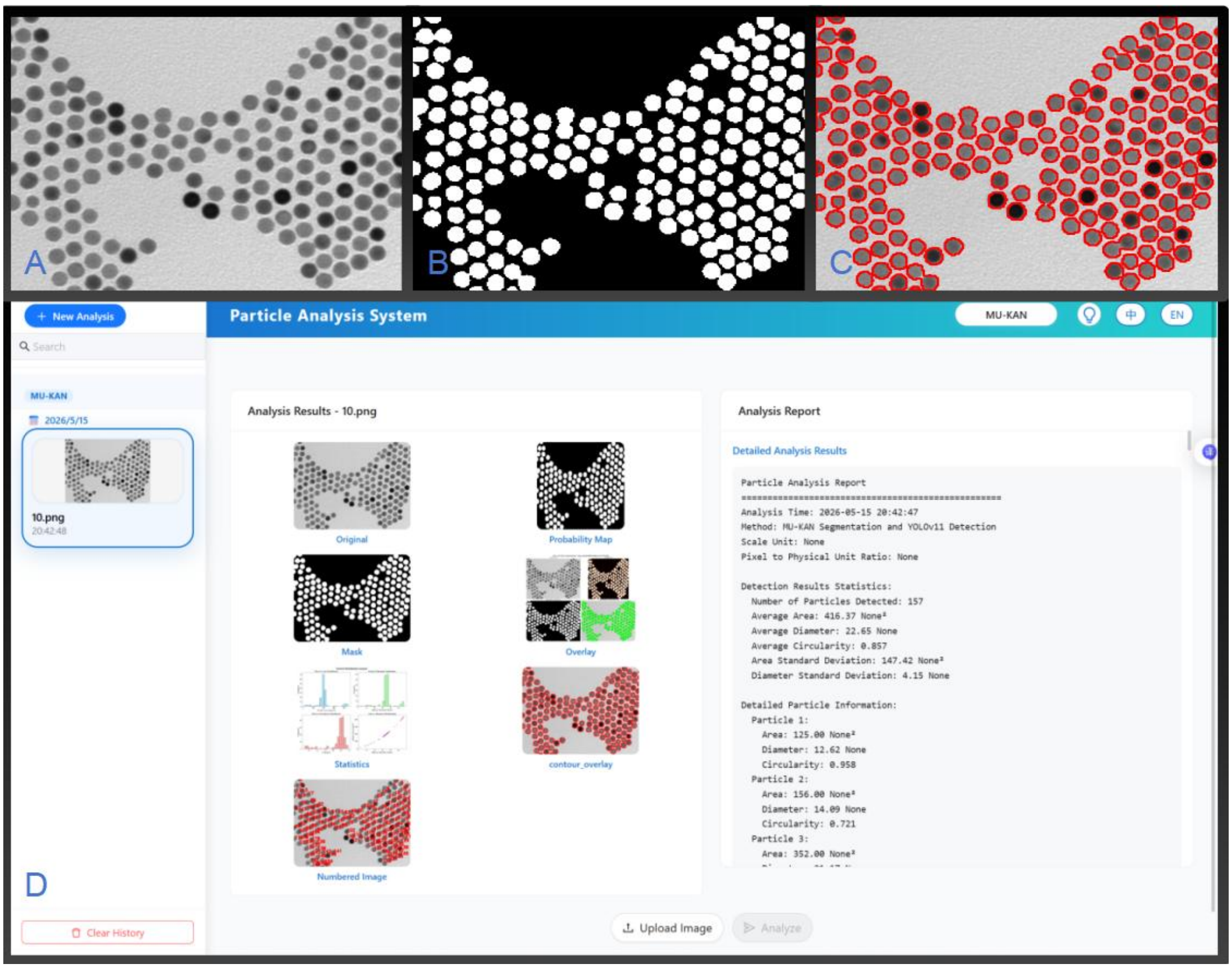

Figure.S11 TEM Example 1.

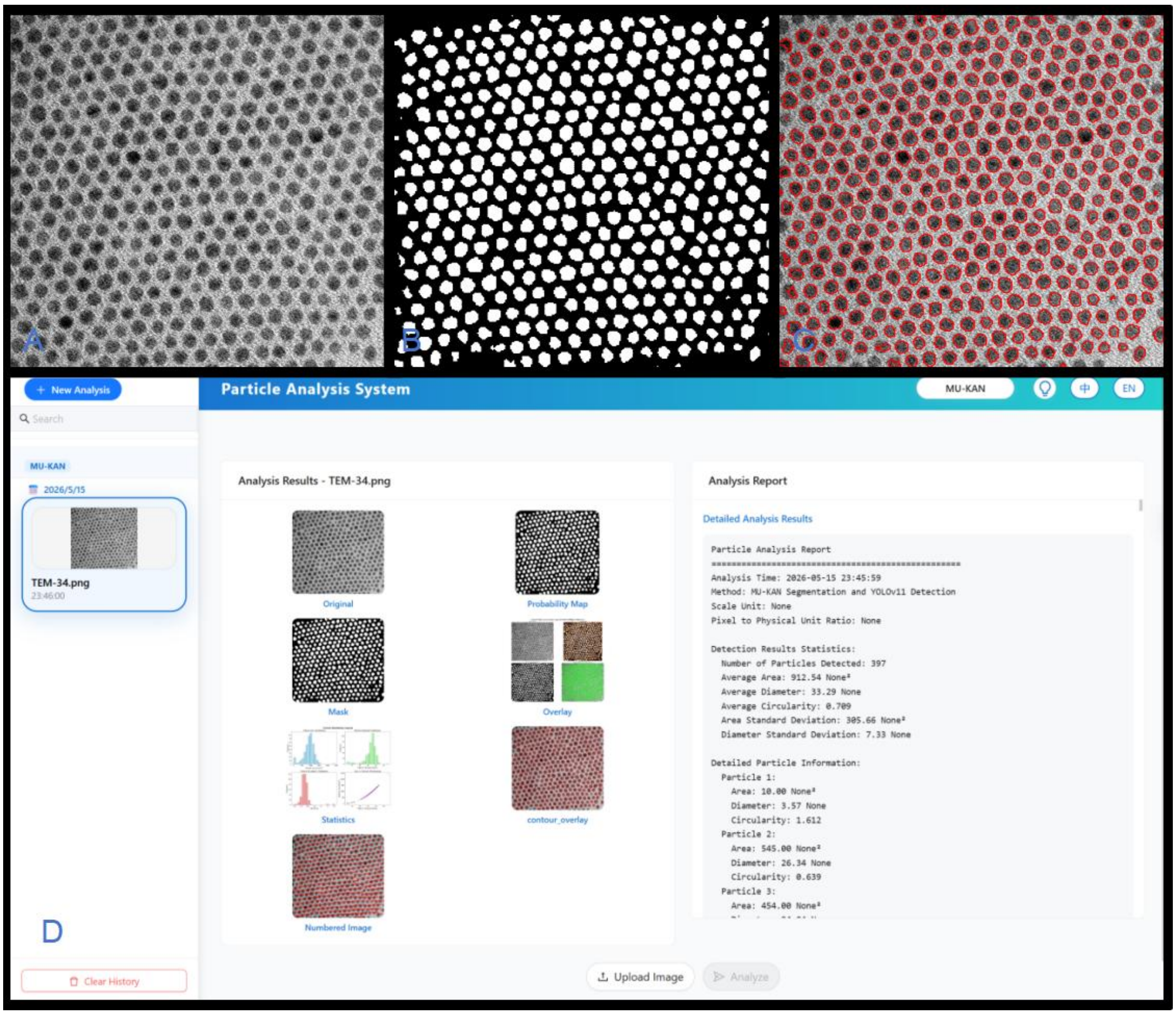

Figure.S12 TEM Example 2.

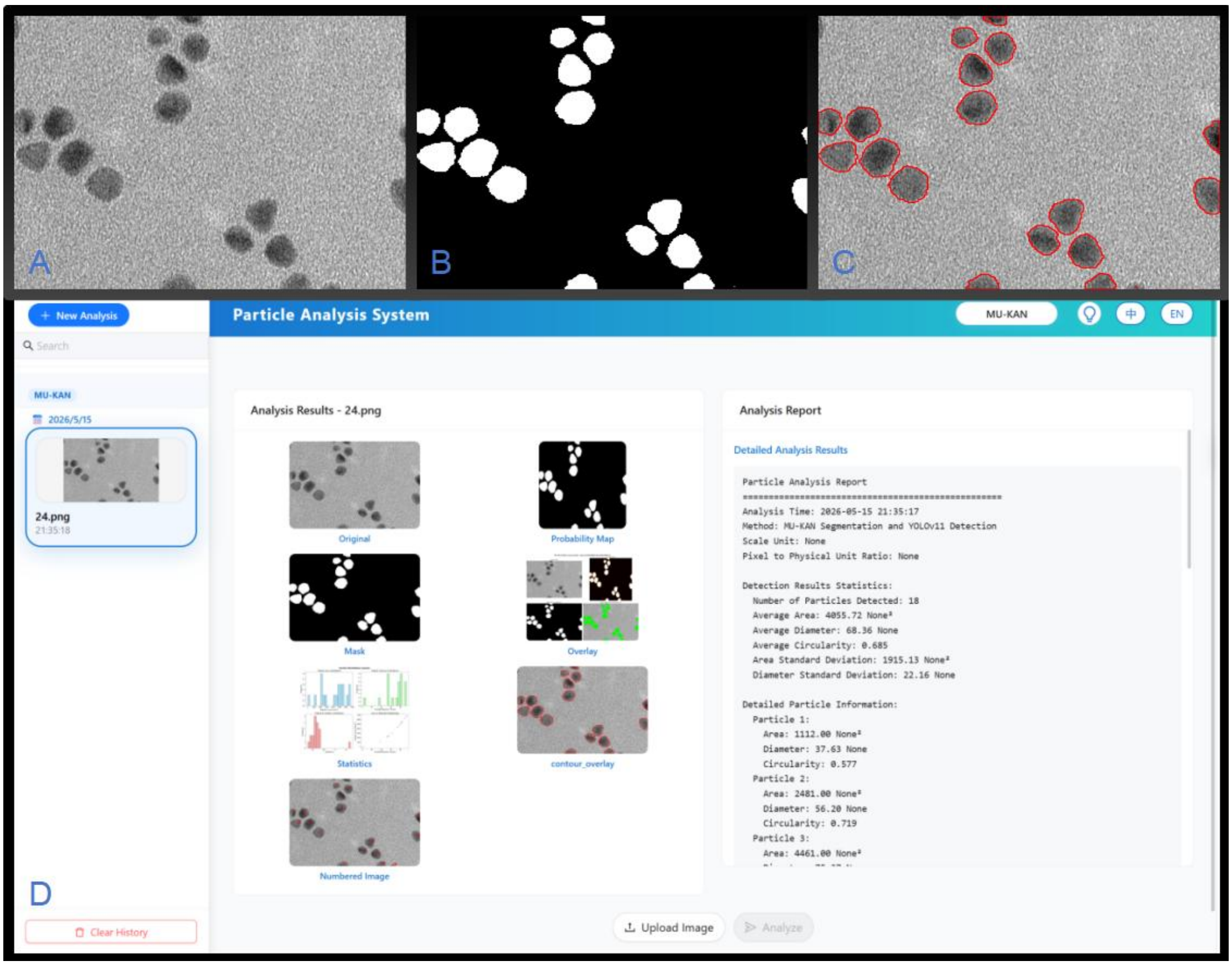

Figure.S13 TEM Example 3.

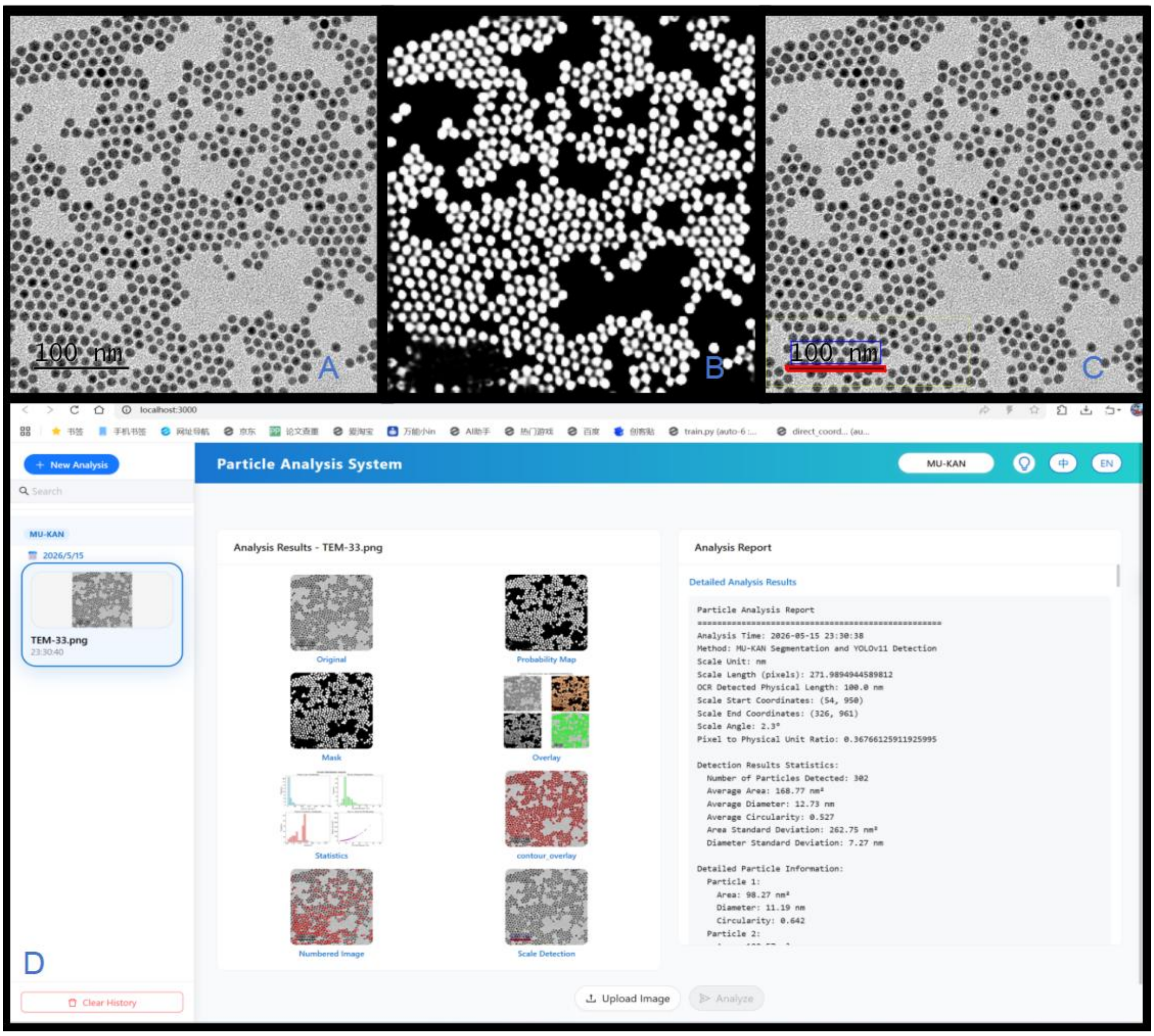

Figure .S14 TEM Example 4.

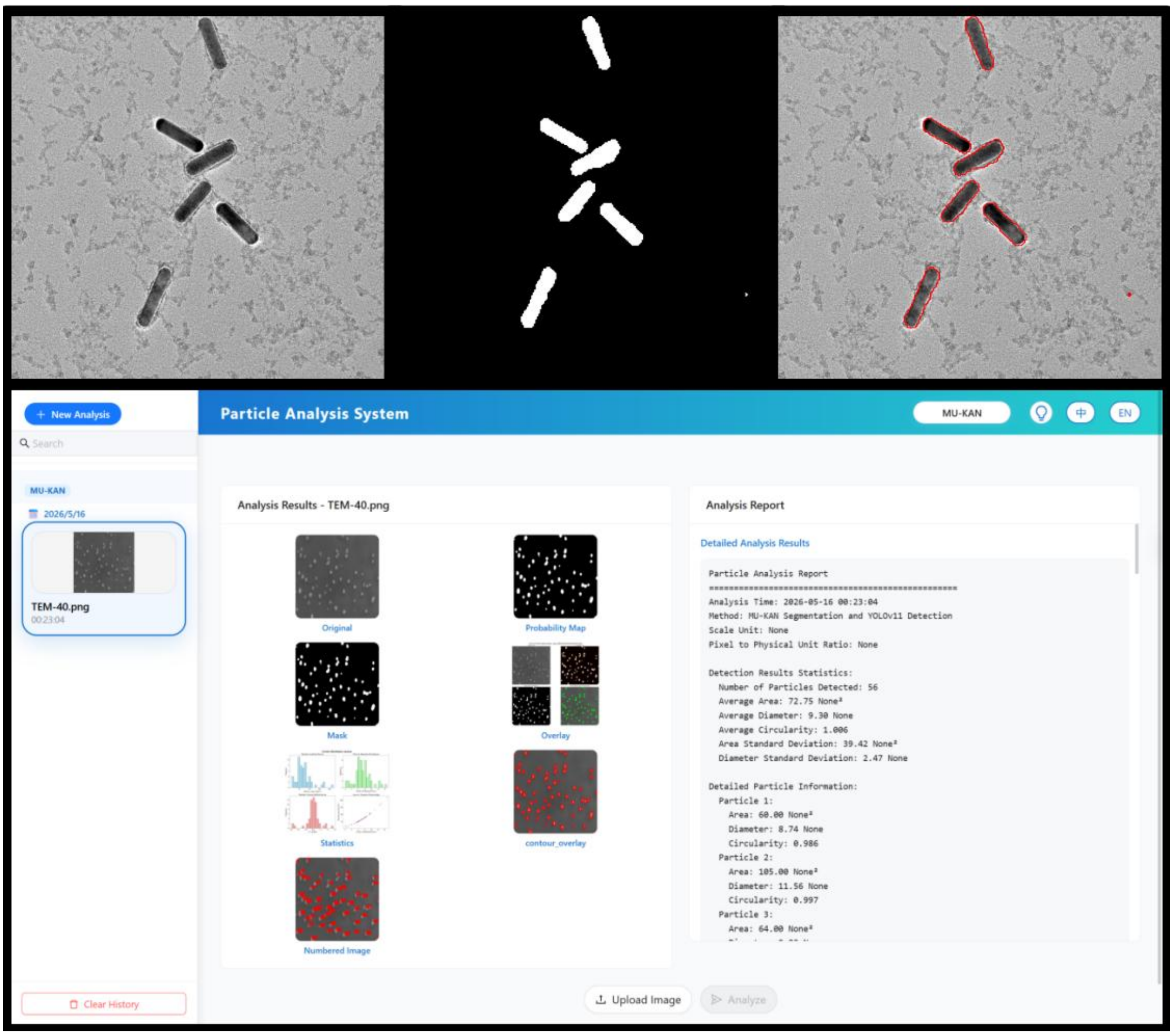

Figure.S15 TEM Example 5.

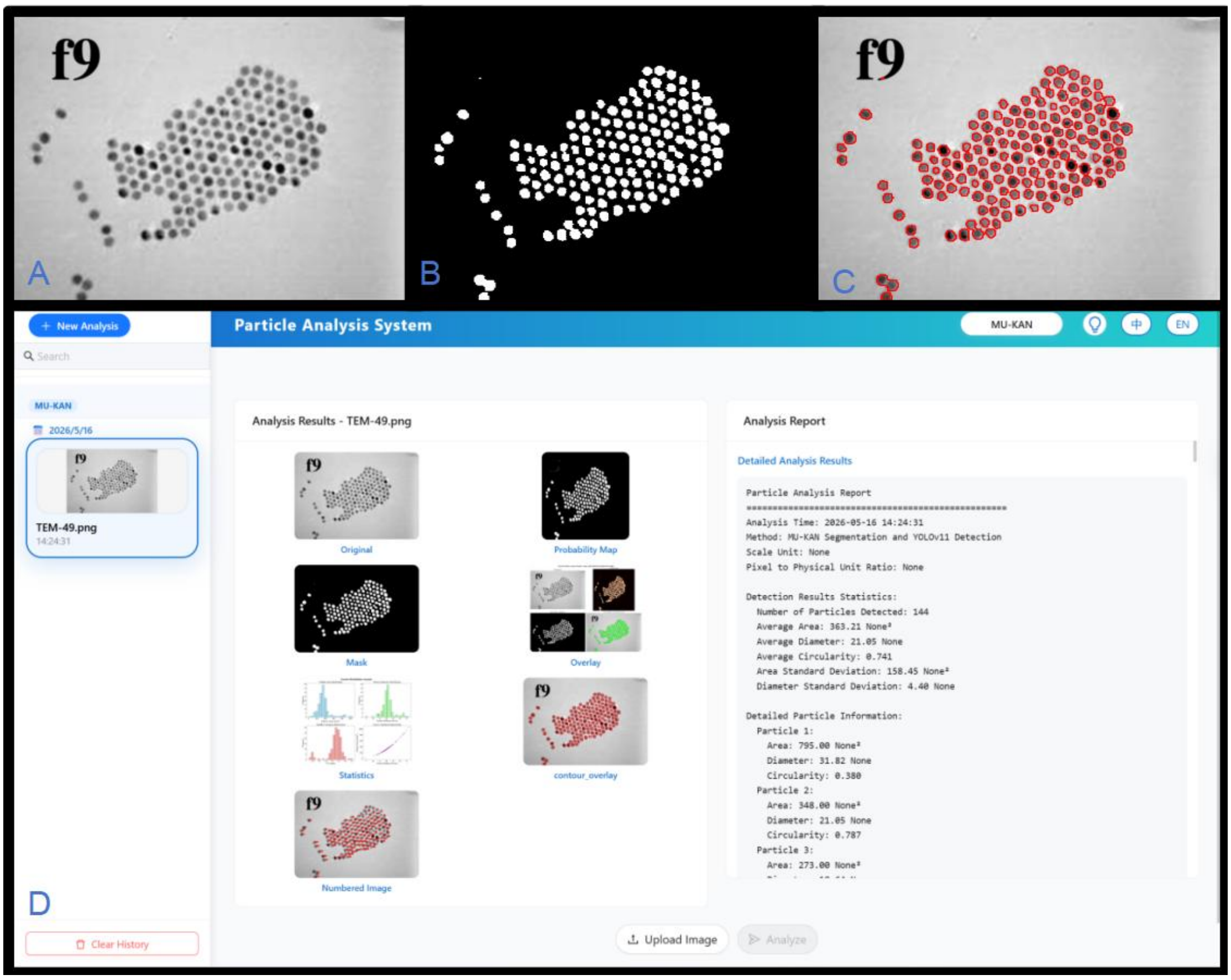

Figure .S16 TEM Example 6.

S4. Additional Loss Function Analysis

To further investigate the influence of loss functions on SEM nanoparticle segmentation, additional experiments were conducted on the NanoSEM-1707 dataset using focal loss under the same dataset split, training configuration, data augmentation strategy, optimization settings, and random seed as those adopted in the main manuscript.

The quantitative results are summarized in Table S2. Overall, the performance trends observed on NanoSEM-1707 were generally consistent with those obtained on NanoSEM-464. For the proposed MU-KAN framework, replacing BCE loss with focal loss resulted in a slight decrease in segmentation performance, with the IoU changing from 0.8243 to 0.8197 and the F1-score changing from 0.9029 to 0.8932. Similar trends were observed across most of the evaluated architectures.

Table S2 Benchmark results of different segmentation architectures on NanoSEM-1707 under focal-loss optimization.

Methods	IoU	Loss	F1	Accuracy	Precision
BiSeNetV2[38]	0.4188	0.0296	0.5573	0.9542	0.8060
U-Net (Dense)[39]	0.7919	0.0037	0.8757	0.9829	0.8792
DCSAU-Net[40]	0.7813	0.0063	0.8675	0.9816	0.8454
U-Net(ResUNet)[41]	0.6935	0.0070	0.8039	0.9778	0.9320
U-Net (VGG)[42]	0.7623	0.0065	0.8552	0.9825	0.9230
FPN[43]	0.6054	0.0097	0.7337	0.9700	0.8730
deeplabv3plus[44]	0.6661	0.0075	0.7809	0.9760	0.9158
TransUNet [45]	0.6910	0.0071	0.8011	0.9780	**0.9412**
U-KAN[46]	0.7938	0.0057	0.8768	0.9824	0.8472
MU-KAN	**0.8197**	**0.0036**	**0.8932**	**0.9857**	0.8944

These results suggest that focal loss does not provide a clear and consistent advantage for the segmentation tasks considered in this study. Across the evaluated architectures, most models exhibited either comparable or reduced segmentation performance when focal loss was adopted. Although slight improvements were observed in a limited number of cases, the overall trend did not indicate a consistent benefit across different architectures and datasets. One possible explanation is that SEM nanoparticle images contain numerous ambiguous pixels located near weak-contrast boundaries, noisy regions, and partially agglomerated particles. By assigning greater weights to these hard samples, focal loss may place excessive emphasis on local uncertainties during optimization, which does not necessarily translate into improved global mask overlap and particle delineation. Therefore, BCE loss was retained as the default optimization objective in the proposed framework.

S5. Extended Morphological Characterization of Irregular Particles

Equivalent circular diameter (ECD) is widely used as a standardized area-equivalent size descriptor for nanoparticle characterization. Since the NanoSEM-464 and NanoSEM-1707 benchmark datasets used in this study predominantly contain

approximately circular nanoparticles, ECD was adopted as the primary descriptor for particle-size statistics in the main text.

To further improve the characterization of elongated, anisotropic, and irregular particles, the particle morphology analysis module was extended to calculate additional geometric descriptors. In addition to ECD and circularity, the framework now automatically computes major axis length, minor axis length, aspect ratio, Feret diameter, minimum Feret diameter, solidity, and extent for each detected particle.

The major and minor axis lengths were obtained from ellipse fitting whenever sufficient contour points were available. The larger fitted axis was defined as the major axis, whereas the smaller fitted axis was defined as the minor axis. For particles with insufficient contour points for ellipse fitting, the corresponding dimensions of the bounding rectangle were used as approximations.

The aspect ratio was calculated as:

$$\text{Aspect}=\text{MajorAxis}/\text{MinorAxis} \tag{1}$$

where values close to 1 indicate approximately equiaxed particles, while larger values indicate elongated or rod-like morphologies.

The Feret diameter and minimum Feret diameter were estimated using the minimum-area rotated bounding rectangle:

$$\text{Feret Diameter}=\max\left(W_r, H_r\right) \tag{2}$$

$$\text{Minimum Feret Diameter}=\min\left(W_r, H_r\right) \tag{3}$$

where W_r and H_r denote the width and height of the rotated bounding rectangle, respectively. These descriptors represent the maximum and minimum particle spans.

Solidity was calculated as:

$$\text{Solidity}=A/A_{\text{convex}} \tag{4}$$

where A is the particle area and A_{convex} is the area of the corresponding convex hull. Solidity quantifies the degree of FFFconvexity of a particle and may decrease for particles exhibiting boundary concavities, adhesion, or irregular shapes.

Extent was calculated as:

$$\text{Extent}=A/A_{\text{box}} \tag{5}$$

where A_{box} is the area of the axis-aligned bounding rectangle. Extent describes the proportion of the bounding box occupied by the particle and provides an indication of particle compactness and geometric regularity.

Representative examples of elongated and anisotropic particles and their corresponding morphology measurements are shown in Fig. S17 andFig. S18. In addition to the original equivalent circular diameter (ECD), the framework automatically computes additional geometric descriptors, including major axis length, minor axis length, aspect ratio, Feret diameter, minimum Feret diameter, circularity, and solidity. These descriptors provide complementary information on particle elongation, anisotropy, compactness, and deviations from circular morphology, enabling a more comprehensive characterization of particle geometry than ECD alone.

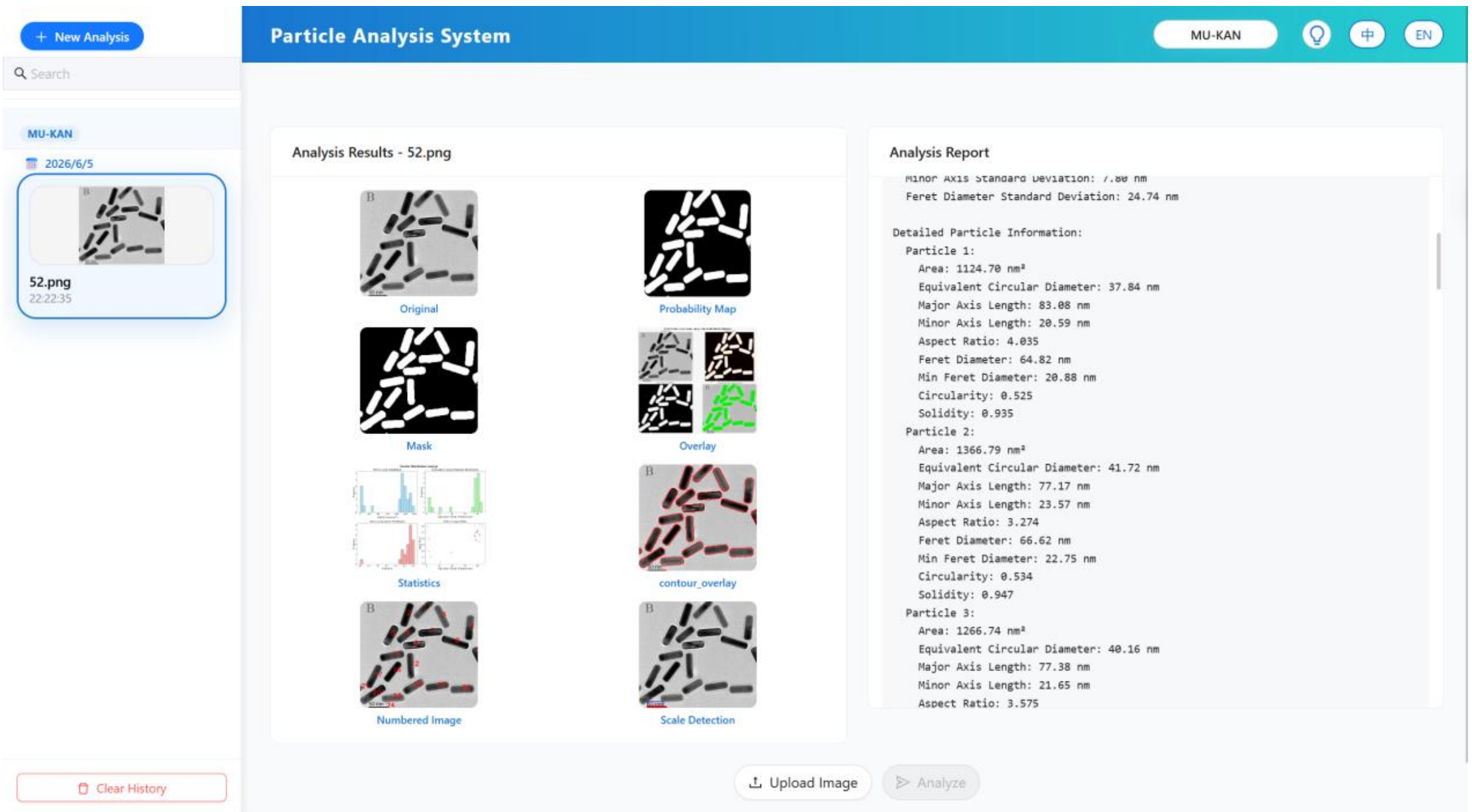

Figure.S17 Non-Circular Particle Example 1.

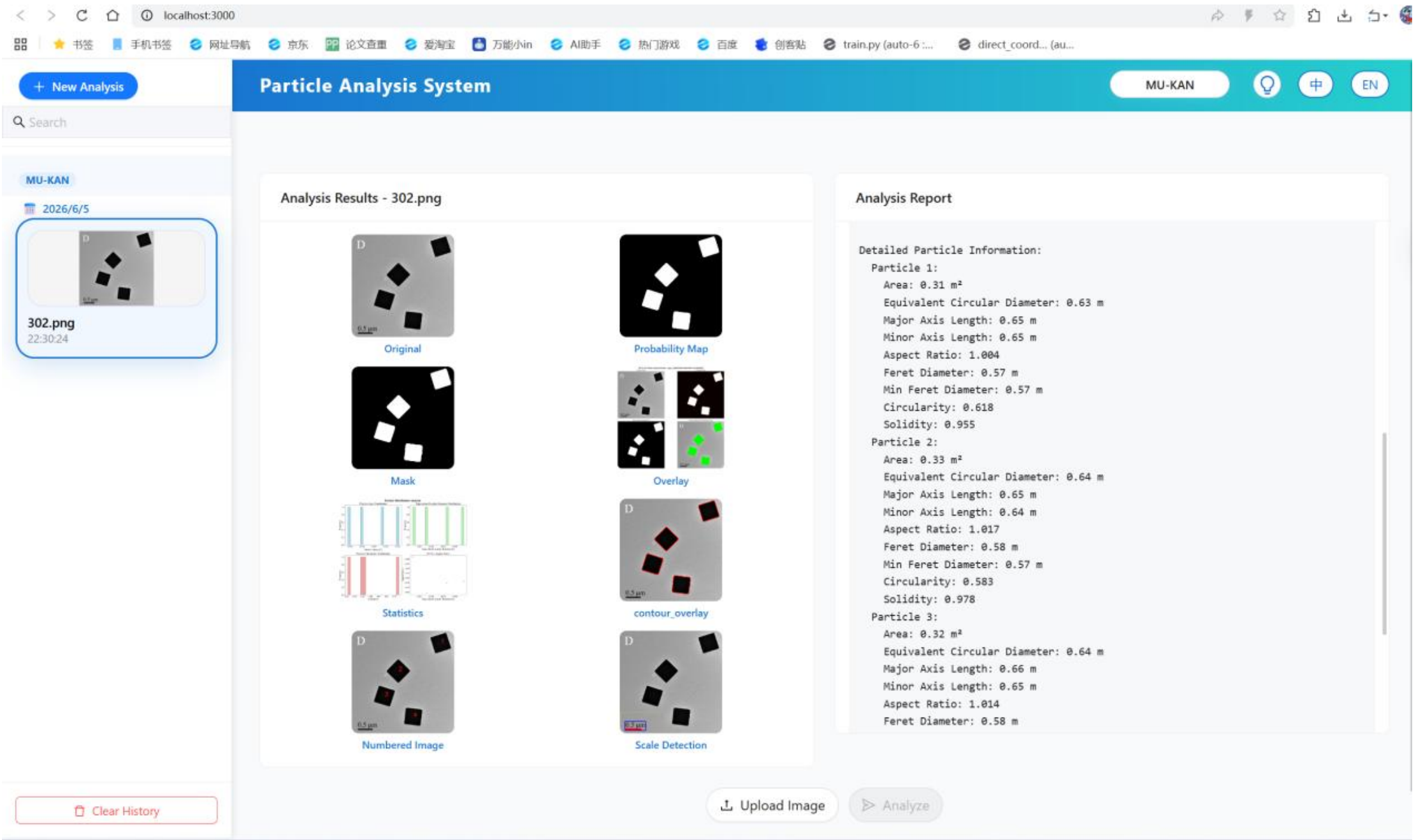

Figure.S18 Non-Circular Particle Example 2.

S6. Validation of Particle Size Measurement Accuracy

To quantitatively evaluate the accuracy of the particle measurement module, the geometric parameters obtained by the proposed MU-KAN framework were compared with those measured using ImageJ, a widely used software for nanoparticle morphology characterization.

Four representative SEM images (S20, S22, S24, and S26) containing nanoparticles with different particle distributions, orientations, and morphological characteristics were selected for quantitative evaluation. For each image, all detectable particles were measured using both MU-KAN and ImageJ, and the resulting geometric descriptors were subsequently compared.

Five commonly used geometric descriptors were evaluated: particle area, major axis length, minor axis length, Feret diameter, and minimum Feret diameter. Scatter plots comparing the measurements obtained by MU-KAN and ImageJ are presented as R^2 correlation graphs, and the mean relative errors (MRE) were calculated to quantify measurement deviation.(Note: As ImageJ was used as the baseline, the software may occasionally misidentify scale bars as particles. Therefore, this analysis only considers true particles, and particle numbering may not start from 1 and may be discontinuous.

Table S3 Particle size measurements obtained by ImageJ and MU-KAN for SEM Image S20.

Methods	Particle Number	Area	Major	Minor	Feret	MinFeret
	1	1425.771	75.388	24.08	69.857	22.798

ImageJ	5	1216.536	74.369	20.828	69.167	20.281
	8	1390.279	75.821	23.347	69.811	22.161
	9	1232.085	65.381	23.994	61.157	22.932
	10	1155.016	66.479	22.121	61.323	20.882
	11	1306.449	73.984	22.484	68.437	21.064
	12	1416.306	85.864	21.002	78.744	19.962
	13	1135.411	66.068	21.881	62.47	21.213
	14	1347.688	74.864	22.921	68.755	21.608
	15	1255.746	78.424	20.388	71.797	19.566
	16	1237.493	72.463	21.744	66.777	20.793
	17	1326.055	77.323	21.836	71.608	20.569
	18	1179.692	68.158	22.037	63.319	20.787
	19	1194.565	71.718	21.208	65.906	20.286
	20	1563.345	86.837	22.922	79.768	21.868
	21	1288.196	71.807	22.842	66.047	21.669
	22	1065.779	67.389	20.137	63.694	20.307
MU-KAN	1	1409.82	74.59	23.64	69.34	22.39
	5	1249.15	75.10	21.22	69.75	20.72
	8	1381.94	74.94	22.82	69.08	21.55
	9	1269.75	66.12	24.44	61.61	23.38
	10	1139.26	65.76	21.62	60.83	20.37
	11	1346.28	74.82	22.93	69.09	21.55
	12	1370.61	84.75	20.55	77.95	19.47
	13	1174.95	66.81	22.32	62.98	21.73
	14	1305.44	74.22	22.41	68.16	21.07
	15	1213.52	77.62	19.97	71.11	19.11
	16	1276.04	73.18	22.19	67.32	21.24
	17	1285.91	76.44	21.39	70.90	20.05
	18	1214.06	68.90	22.48	63.91	21.29
	19	1157.12	70.92	20.70	65.24	19.74
	20	1515.07	85.58	22.37	78.88	21.27
	21	1335.71	72.61	23.34	66.69	22.20
	22	1025.79	66.55	19.71	63.00	19.82

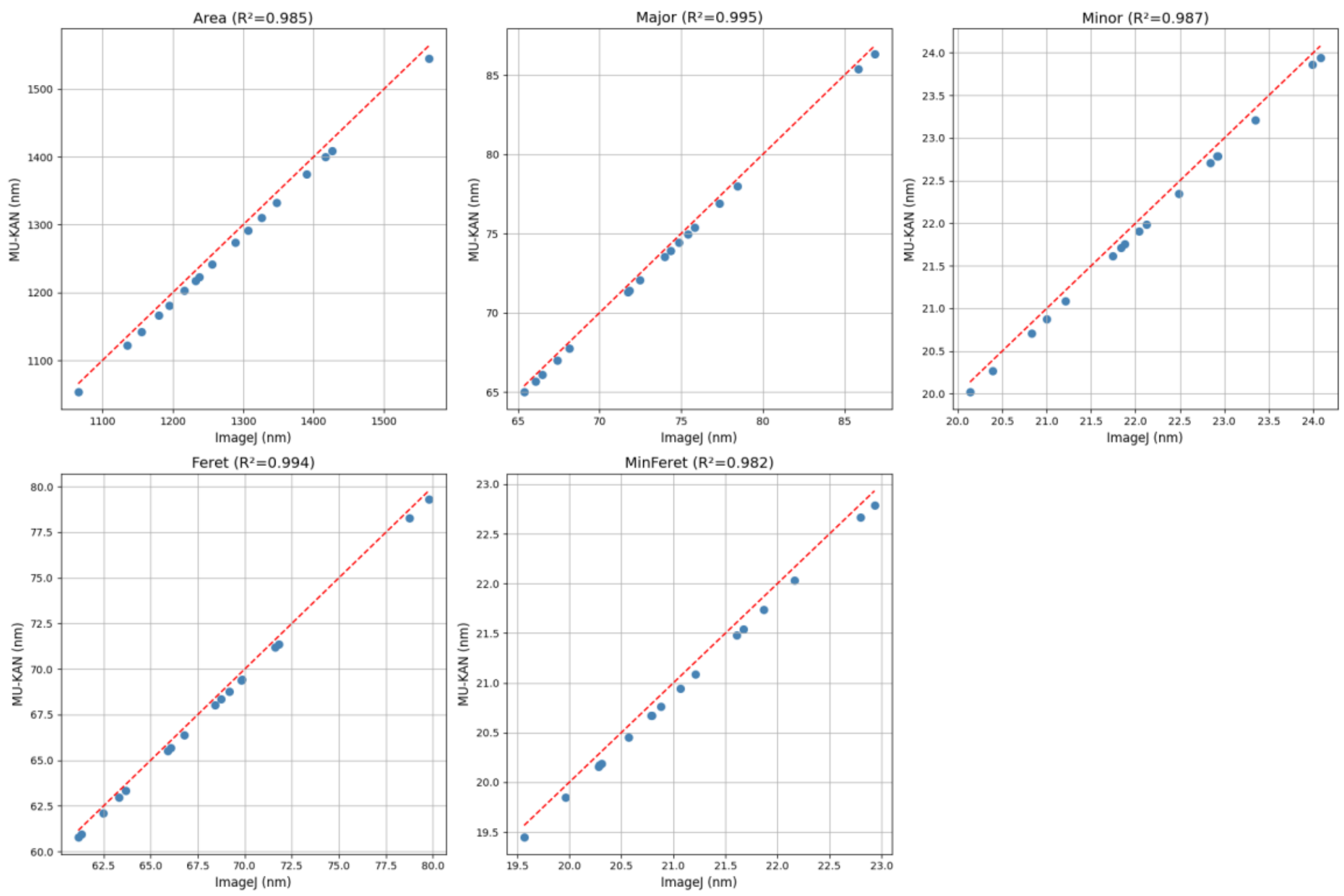

Figure.S19 Correlation analysis between MU-KAN and ImageJ measurements for SEM Image S20.

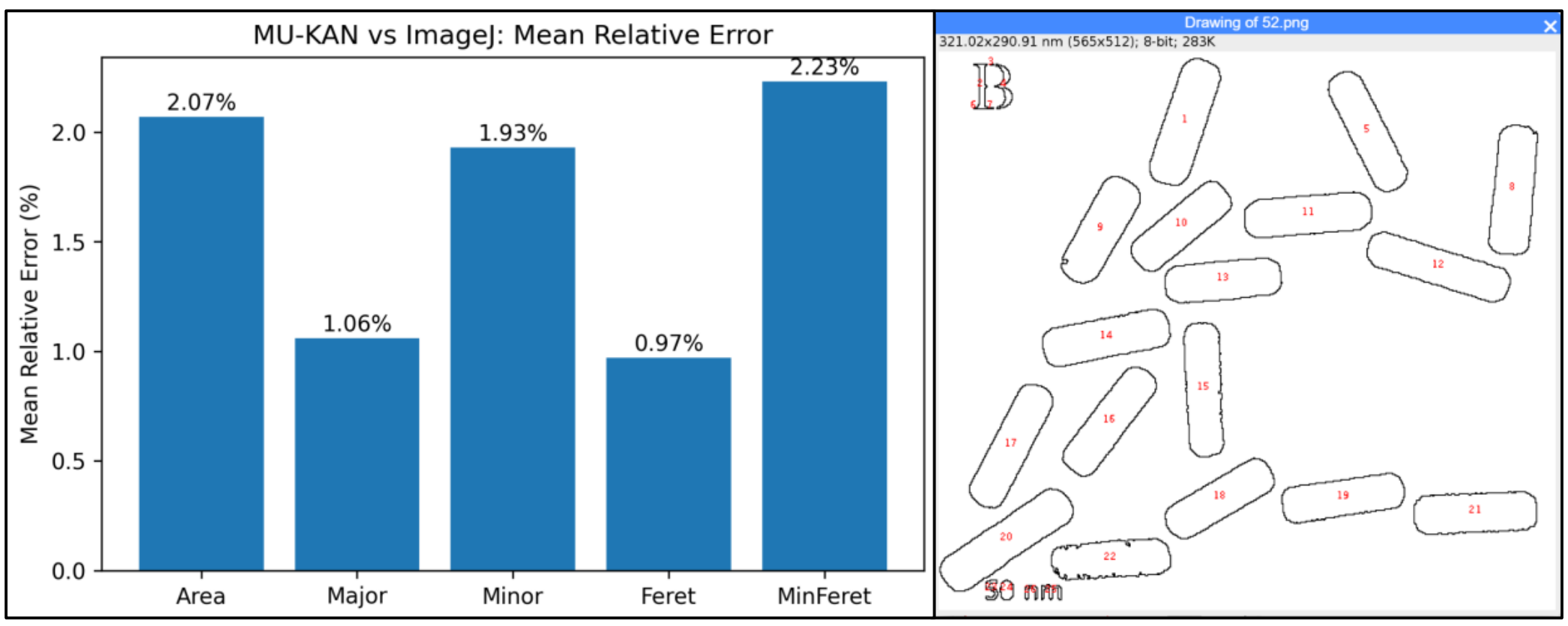

Figure.S20 Quantitative comparison between MU-KAN and ImageJ measurements for SEM Image . The left panel shows the mean relative errors (MRE) of different geometric descriptors, while the right panel illustrates the particle numbering generated by ImageJ for particle correspondence.

Table S4 Particle size measurements obtained by ImageJ and MU-KAN for SEM Image S22.

Methods	Particle Number	Area	Major	Minor	Feret	MinFeret
	3	61.922	9.019	8.742	9.65	8.894
	4	89.365	10.999	10.345	11.43	10.589
	5	74.002	10.182	9.253	10.748	9.422
	6	113.407	12.112	11.921	12.708	11.986
	7	76.817	10.121	9.664	10.726	9.589
	8	72.126	9.654	9.512	10.279	9.589
	9	68.842	9.593	9.137	10.118	8.904
	10	77.051	10.107	9.707	10.633	9.796
	11	62.157	9.162	8.638	9.54	8.561
	12	74.588	10.641	8.924	11.218	9.097
	13	83.384	10.471	10.139	10.943	10.274
	14	71.187	9.708	9.337	10.165	9.589
	15	90.655	10.893	10.597	11.507	10.616
	16	75.292	10.006	9.581	10.483	9.589
	17	66.379	9.213	9.174	9.86	9.246
	18	70.015	9.667	9.222	10.182	9.444
	19	83.971	10.566	10.118	11.265	10.261
ImageJ	20	68.607	9.558	9.139	9.984	9.246
	21	76.465	10.047	9.69	10.638	9.897
	22	79.749	10.201	9.954	10.748	9.931
	23	88.31	10.914	10.303	11.512	10.556
	24	61.571	8.988	8.722	9.668	8.904
	25	56.176	8.861	8.072	9.354	8.219
	26	77.755	10.331	9.582	11.218	9.928
	27	77.872	11.132	8.906	13.569	9.246
	28	74.354	9.916	9.547	10.438	9.589
	29	63.212	9.24	8.711	9.668	8.895
	30	71.187	9.593	9.449	10.182	9.589
	31	75.175	9.967	9.603	10.505	9.893
	32	70.484	9.579	9.368	10.002	9.573
	33	76.23	10.177	9.537	10.748	9.589
	34	74.237	9.982	9.469	10.302	9.589
	35	69.428	9.481	9.324	9.86	9.444
	36	78.107	10.423	9.541	10.943	9.589
	37	81.977	10.955	9.528	11.384	9.603
	38	63.095	9.186	8.745	9.595	8.883
	3	63.15	9.12	8.85	9.73	8.97
	4	87.12	10.88	10.21	11.32	10.47
	5	75.89	10.27	9.34	10.82	9.51
	6	110.02	11.98	11.75	12.58	11.83
	7	78.53	10.22	9.78	10.81	9.67
	8	70.35	9.57	9.42	10.19	9.51

MU-KAN	9	70.11	9.68	9.22	10.20	8.99
	10	79.24	10.21	9.81	10.73	9.89
	11	60.98	9.09	8.55	9.47	8.49
	12	76.21	10.72	9.01	11.30	9.18
	13	81.57	10.38	10.05	10.86	10.19
	14	72.89	9.79	9.42	10.24	9.67
	15	93.01	10.98	10.70	11.60	10.70
	16	73.58	9.92	9.49	10.4	9.51
	17	67.92	9.29	9.26	9.94	9.33
	18	68.54	9.58	9.13	10.10	9.36
	19	86.13	10.65	10.21	11.35	10.35
	20	70.24	9.64	9.22	10.06	9.33
	21	74.82	9.96	9.60	10.56	9.81
	22	81.37	10.29	10.04	10.83	10.02
	23	86.04	10.82	10.19	11.4	10.44
	24	62.91	9.07	8.81	9.75	8.99
	25	57.42	8.94	8.16	9.43	8.30
	26	79.41	10.42	9.67	11.30	10.01
	27	76.15	11.05	8.82	13.45	9.17
	28	76.02	10.00	9.63	10.52	9.67
	29	64.53	9.33	8.80	9.75	8.98
	30	69.51	9.51	9.36	10.10	9.51
	31	73.45	9.88	9.52	10.42	9.81
	32	72.03	9.66	9.45	10.08	9.65
	33	74.51	10.09	9.45	10.66	9.51
	34	75.98	10.07	9.56	10.39	9.67
	35	71.02	9.57	9.41	9.94	9.52
	36	76.45	10.34	9.46	10.86	9.51
	37	80.21	10.87	9.44	11.30	9.53
	38	64.58	9.27	8.83	9.68	8.97

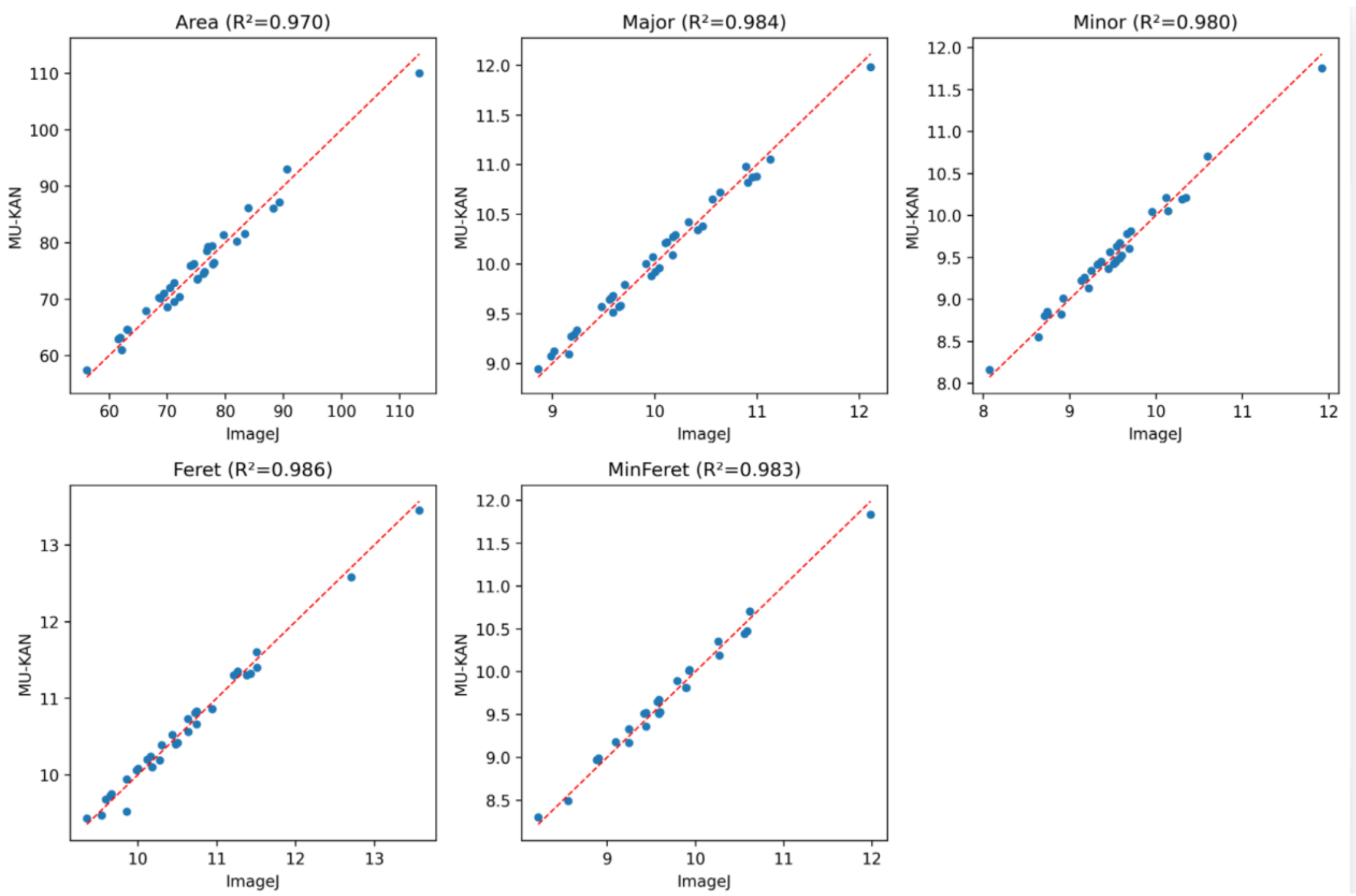

Figure.S21 Correlation analysis between MU-KAN and ImageJ measurements for SEM Image S22.

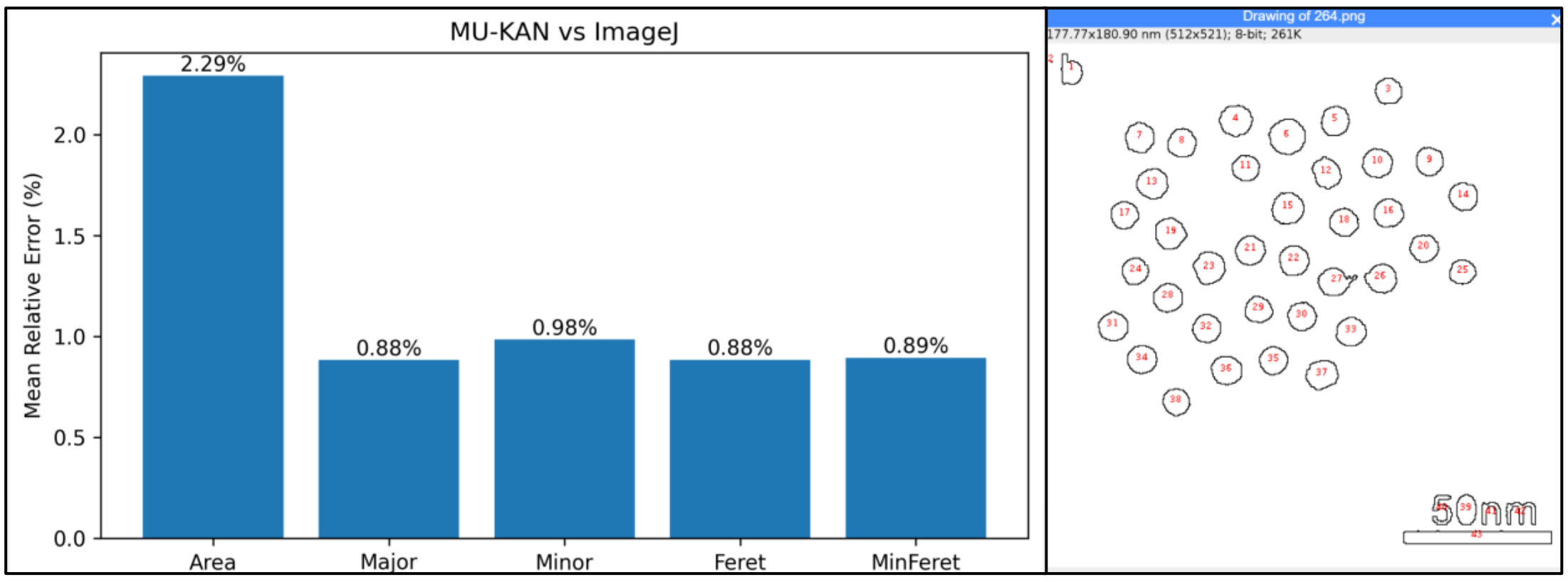

Figure.S22 Quantitative comparison between MU-KAN and ImageJ measurements for SEM Image . The left panel shows the mean relative errors (MRE) of different geometric descriptors, while the right panel illustrates the particle numbering generated by ImageJ for particle correspondence.

Table S5 Particle size measurements obtained by ImageJ and MU-KAN for SEM Image S24.

Methods	Particle Number	Area	Major	Minor	Feret	MinFeret
ImageJ	4	736.016	31.163	30.072	34.177	30.732
	5	697.632	30.919	28.729	34.636	30.751
	8	967.068	38.220	32.216	41.606	33.480
	4	751.23	31.35	30.38	34.39	31.02

MU-KAN	5	682.41	30.72	28.45	34.35	30.47
	8	998.57	38.51	32.59	41.98	33.81

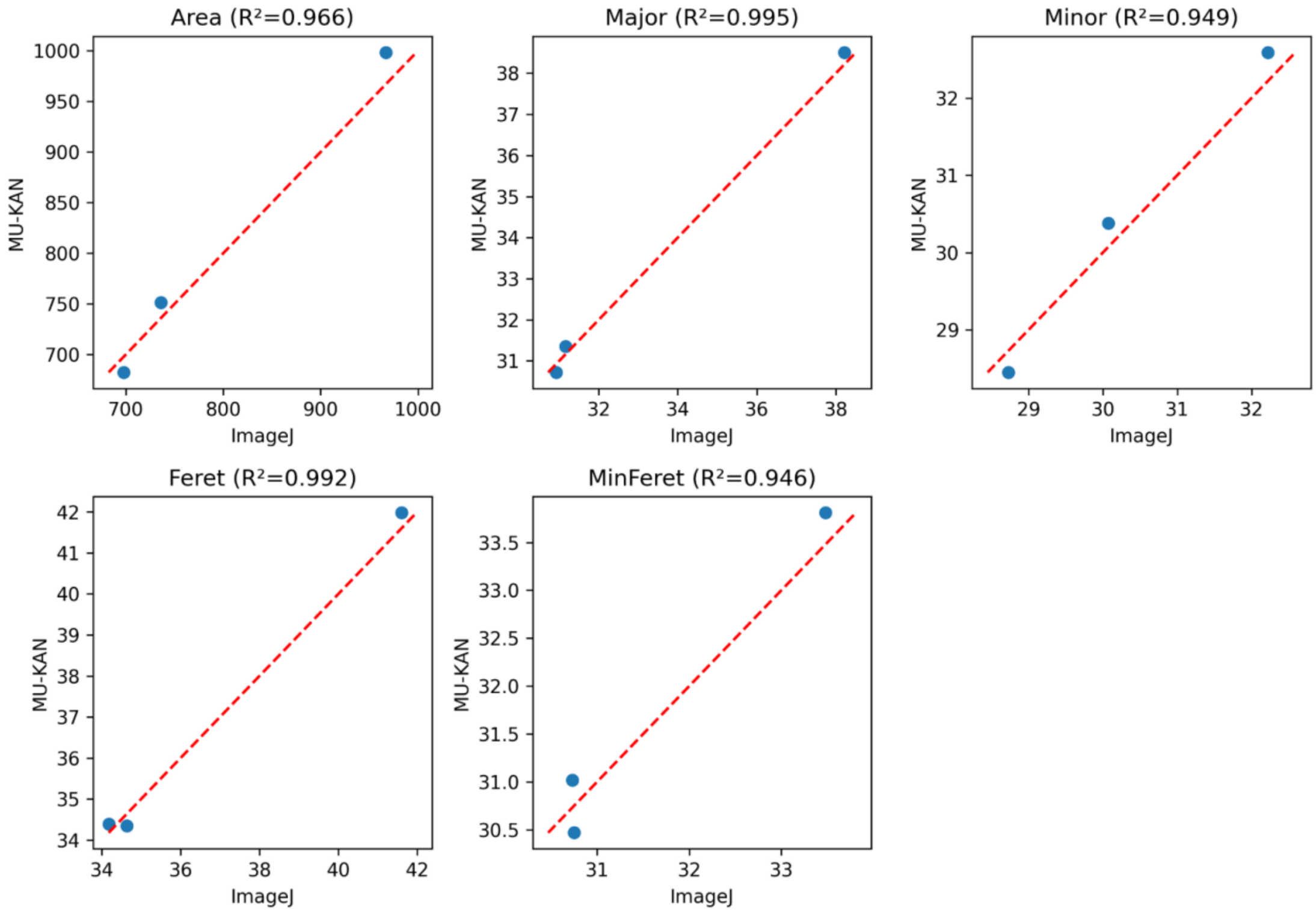

Figure.S23 Correlation analysis between MU-KAN and ImageJ measurements for SEM Image S24.

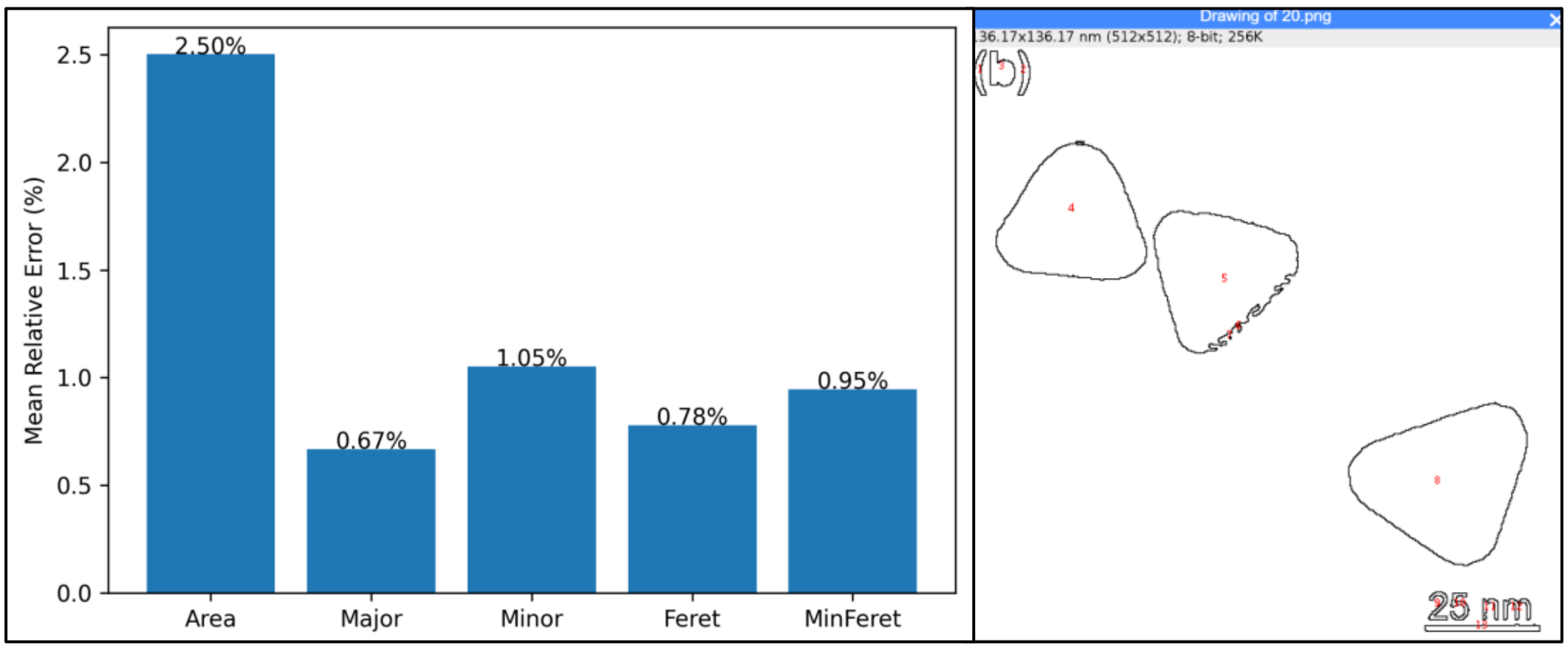

Figure.S24 Quantitative comparison between MU-KAN and ImageJ measurements for SEM Image . The left panel shows the mean relative errors (MRE) of different geometric descriptors, while the right panel illustrates the particle numbering generated by ImageJ for particle correspondence.

Table S6 Particle size measurements obtained by ImageJ and MU-KAN for SEM Image S26.

Methods	Particle Number	Area	Major	Minor	Feret	MinFeret

ImageJ	2	156.894	14.837	13.464	15.113	13.767
	3	193.043	17.967	13.680	18.378	14.050
	5	140.945	14.138	12.693	14.482	12.900
	6	156.200	14.624	13.599	15.125	13.545
	8	161.193	15.057	13.631	15.240	13.545
	9	138.218	14.366	12.250	14.810	12.581
MU-KAN	2	160.78	14.91	13.58	15.16	13.85
	3	189.21	17.89	13.77	18.45	14.12
	5	138.12	14.22	12.59	14.41	12.81
	6	159.74	14.7	13.72	15.19	13.63
	8	164.53	15.14	13.51	15.30	13.64
	9	135.07	14.30	12.34	14.76	12.67

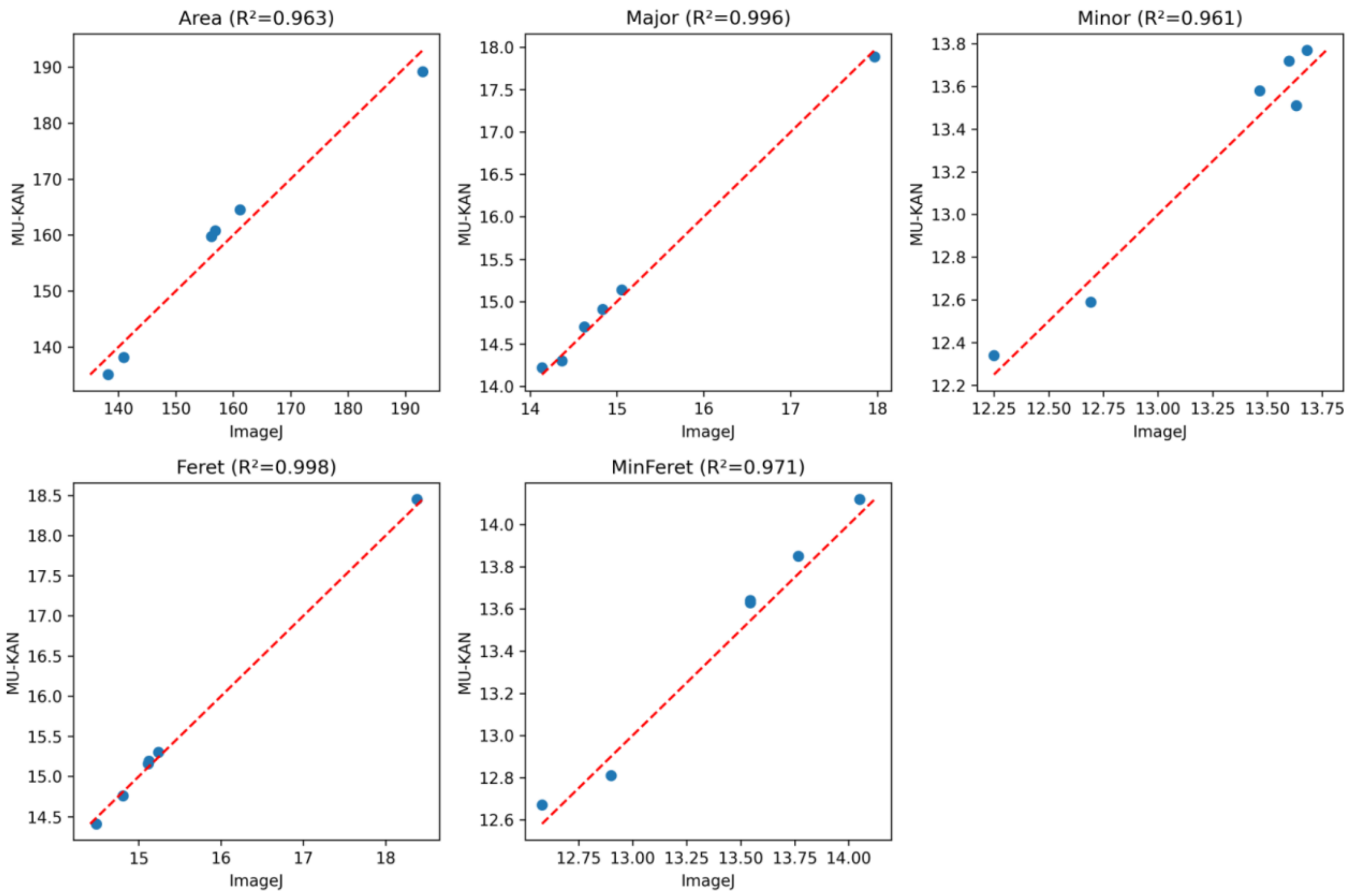

Figure.S25 Correlation analysis between MU-KAN and ImageJ measurements for SEM Image S26.

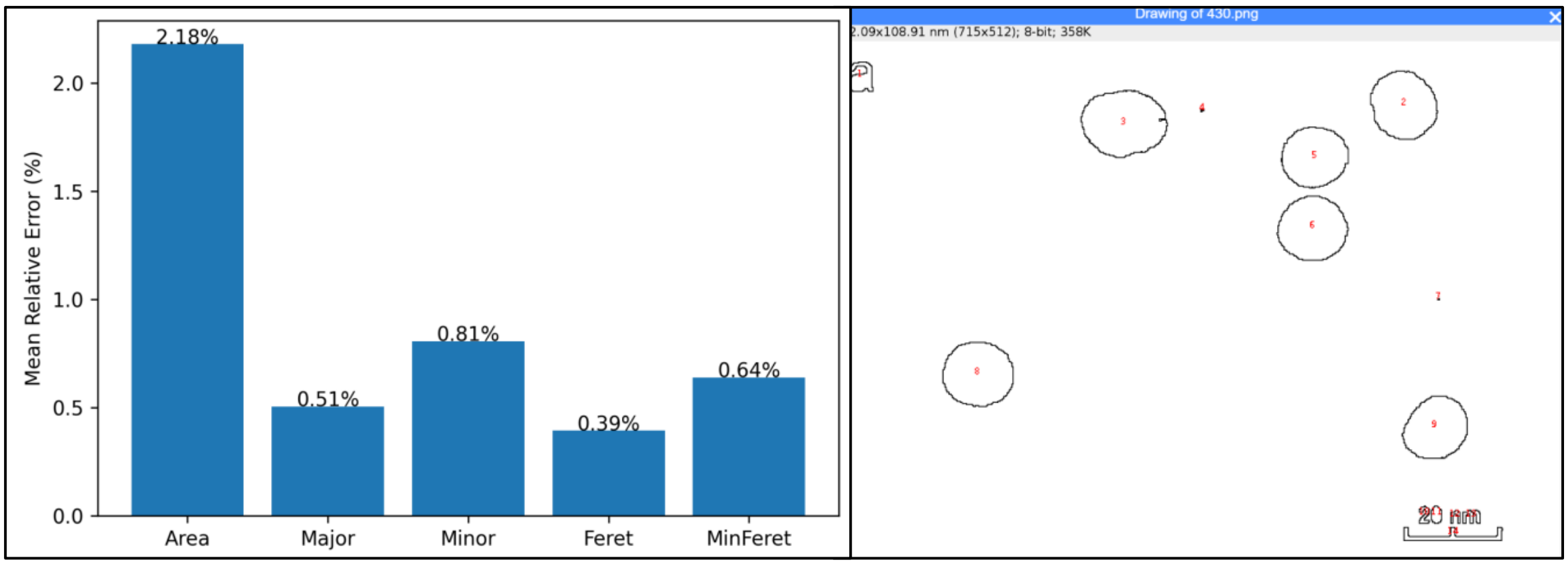

Figure.S26 Quantitative comparison between MU-KAN and ImageJ measurements for SEM Image . The left panel shows the mean relative errors (MRE) of different geometric descriptors, while the

right panel illustrates the particle numbering generated by ImageJ for particle correspondence.

To further quantify measurement deviation, the mean relative error (MRE) and coefficient of determination (R^2) werecalculated as follows:

$$\mathrm{MRE}(\%)=\frac{1}{\mathrm{N}}\sum_{\mathrm{i=1}}^{\mathrm{N}}\frac{|\mathrm{M_i}\text{-}\mathrm{R_i}|}{\mathrm{R_i}}\times 100 \tag{6}$$

where $\mathrm{M_i}$ and $\mathrm{R_i}$ denote the measurements obtained by MU-KAN and ImageJ, respectively.

$$\mathrm{R}^2=1\text{-}\frac{\sum_{\mathrm{i=1}}^{\mathrm{N}}\left(\mathrm{X_{ImageJ,i}}\text{-}\widehat{\mathrm{X}}_{\mathrm{i}}\right)^2}{\sum_{\mathrm{i=1}}^{\mathrm{N}}\left(\mathrm{X_{ImageJ,i}}\text{-}\bar{\mathrm{X}}\right)^2} \tag{7}$$

where$\widehat{\mathrm{X}}_{\mathrm{i}}$ is the fitted value from the linear regression between MU-KAN and ImageJ data.

The coefficients of determination (R^2) for each descriptor demonstrate strong linear correlations between MU-KAN and ImageJ measurements. The mean relative errors (MRE) indicate high measurement accuracy, confirming that MU-KAN provides results consistent with ImageJ across nanoparticles with different distributions and morphologies.

S7. Hyperparameter Optimization and Five-Fold Cross-Validation

To determine an appropriate training configuration for the proposed MU-KAN framework, hyperparameter optimization was performed using the Optuna framework. A total of 30 independent trials were conducted, and the search space included the backbone learning rate, batch size, weight decay, KAN learning rate, KAN weight decay, learning-rate scheduler, and MOGA module configuration. The validation IoU was used as the optimization objective.

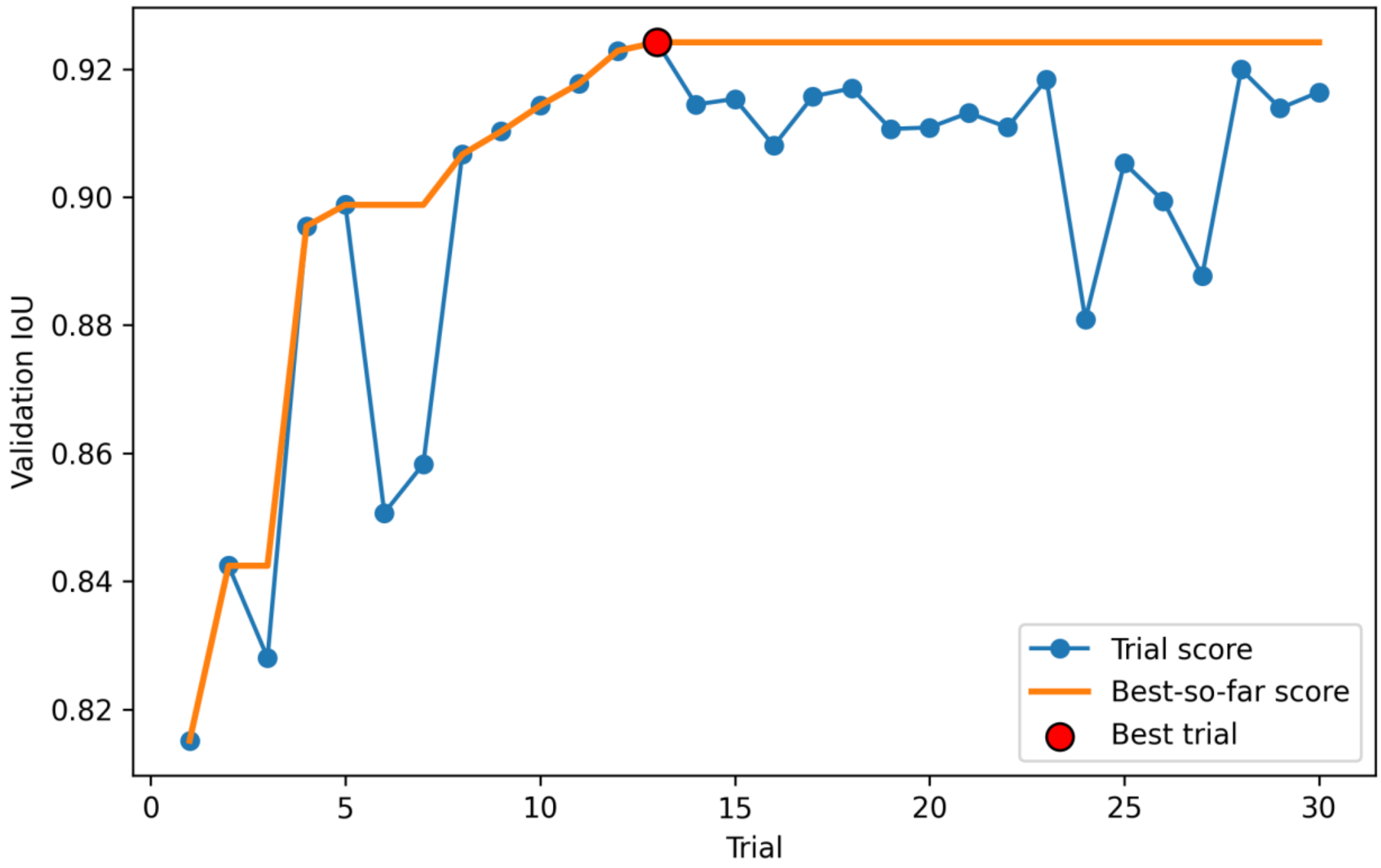

Figure.S27 Optimization History of Hyperparameter Search

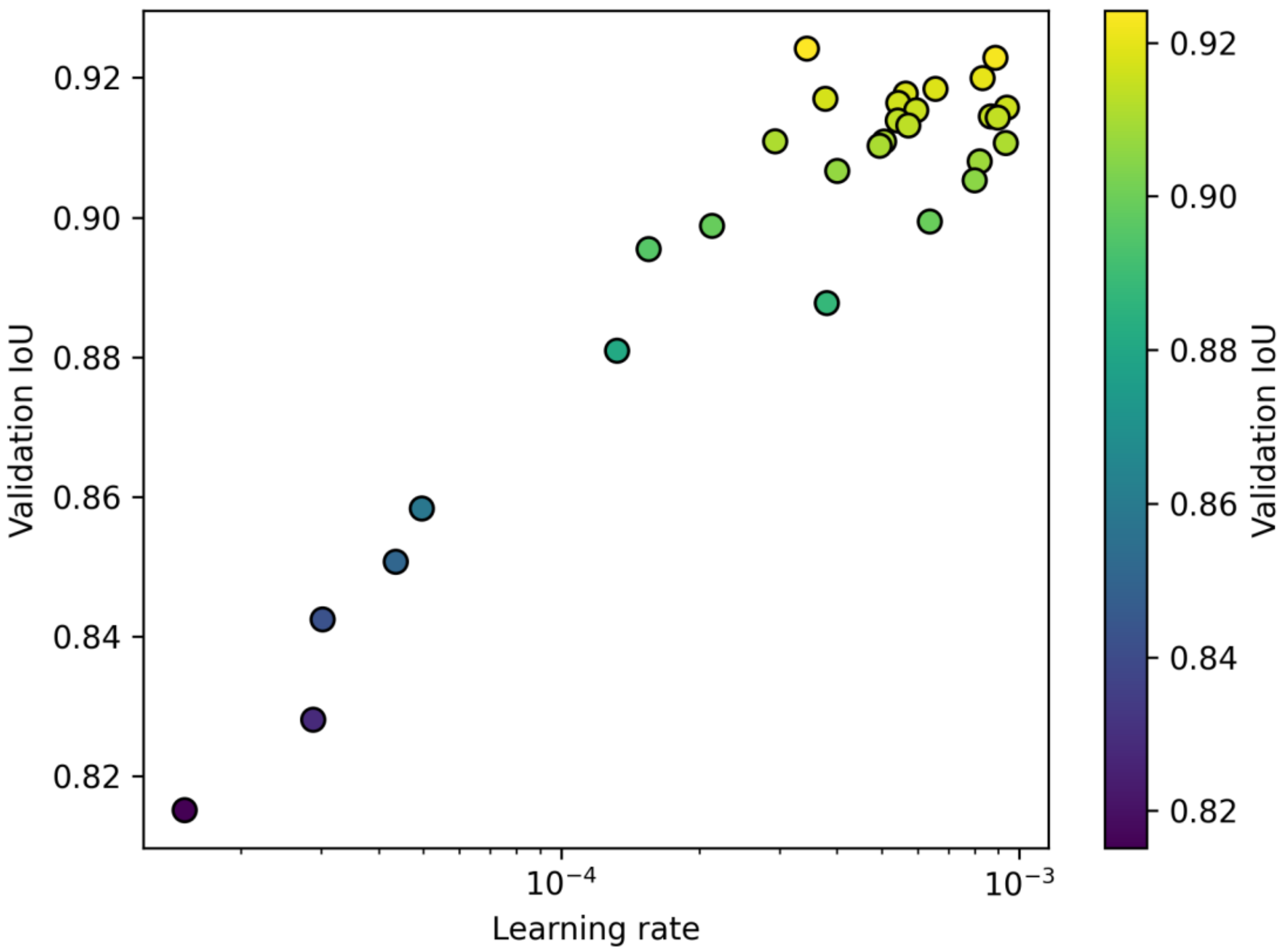

Figure.S28 Relationship Between Learning Rate and Validation IoU

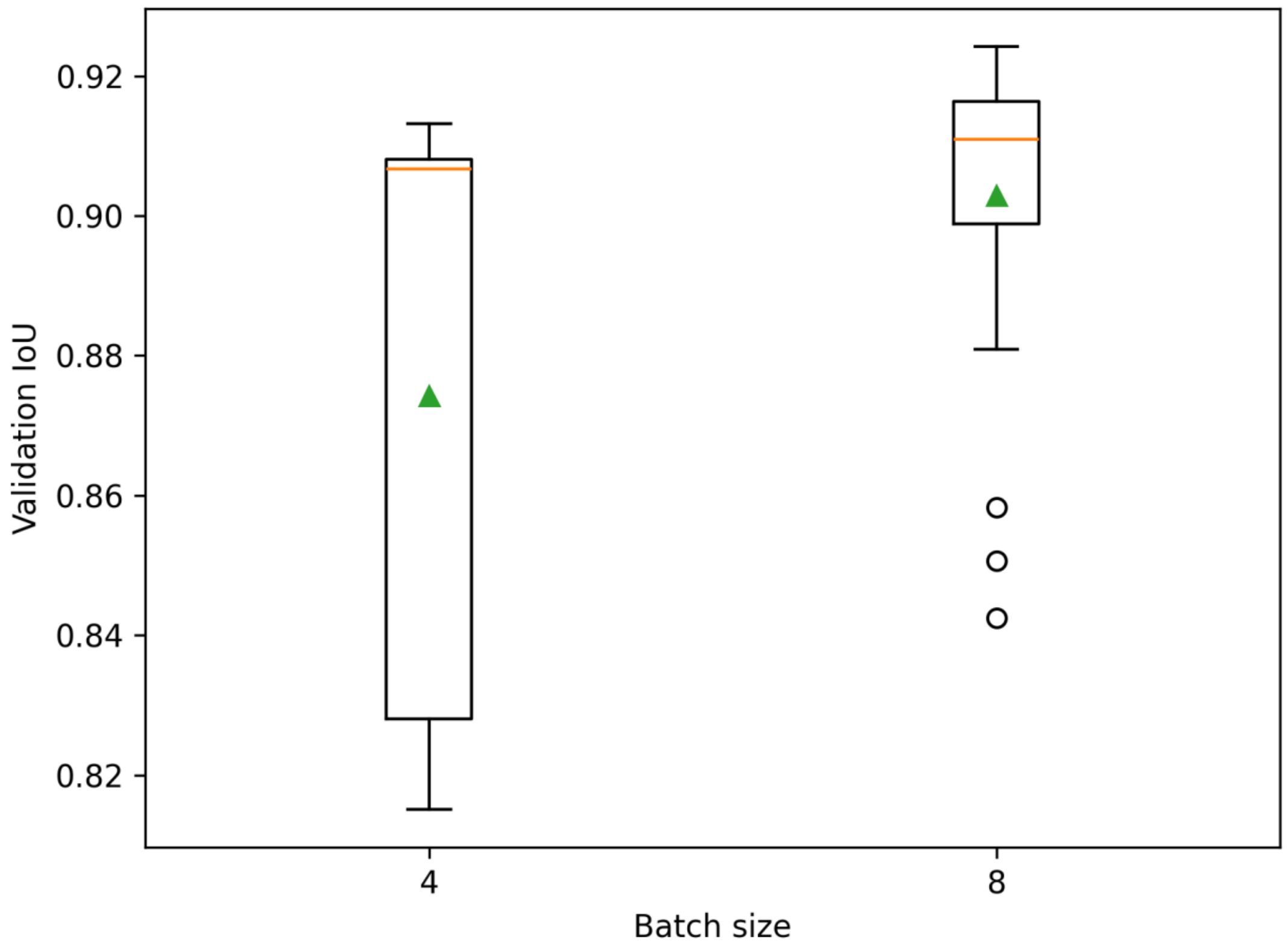

Figure.S29 Comparison of Validation IoU Under Different Batch Sizes

Figure S27 illustrates the optimization history of the Optuna search process. The validation IoU improved progressively during optimization and converged after approximately 13 trials, indicating that a stable optimum had been identified. Figure S28 shows the relationship between backbone learning rate and validation IoU, demonstrating that the best-performing trials were concentrated around the 10^{-4} learning-rate range. The optimal trial yielded a backbone learning rate of approximately 3.44×10^{-4}. Considering that model performance showed limited sensitivity to small perturbations around the optimum, a learning rate of 3×10^{-4} was ultimately adopted for all experiments to improve reproducibility and facilitate implementation. Figure S29 compares the influence of different batch sizes on validation IoU, showing that a batch size of 8 consistently outperformed a batch size of 4.

Based on the optimization results, the final training configuration consisted of BCELoss, batch size = 8, backbone learning rate = 3×10^{-4}, KAN learning rate = 1×10^{-5}, weight decay = 1×10^{-4}, KAN weight decay = 1.13×10^{-5}, CosineAnnealingLR scheduler, and the MOGA module enabled.

To further evaluate the robustness and generalization ability of the optimized MU-KAN framework, five-fold cross-validation was conducted on the NanoSEM-464

dataset using the final hyperparameter configuration. For each fold, approximately 80% of the images were used for training and the remaining 20% were used for validation. The same training strategy, data augmentation procedures, loss function, and hyperparameter settings were maintained throughout all folds to ensure fair comparison.

The average segmentation performance obtained from the five folds is summarized in Figure S30. The proposed MU-KAN framework achieved an average IoU of 0.9116 ± 0.0094, Dice coefficient of 0.9531 ± 0.0054, F1-score of 0.9531 ± 0.0054, Recall of 0.9530 ± 0.0097, Precision of 0.9545 ± 0.0051, and Accuracy of 0.9689 ± 0.0040. The relatively small standard deviations indicate that the segmentation performance remained stable across different train-validation partitions.

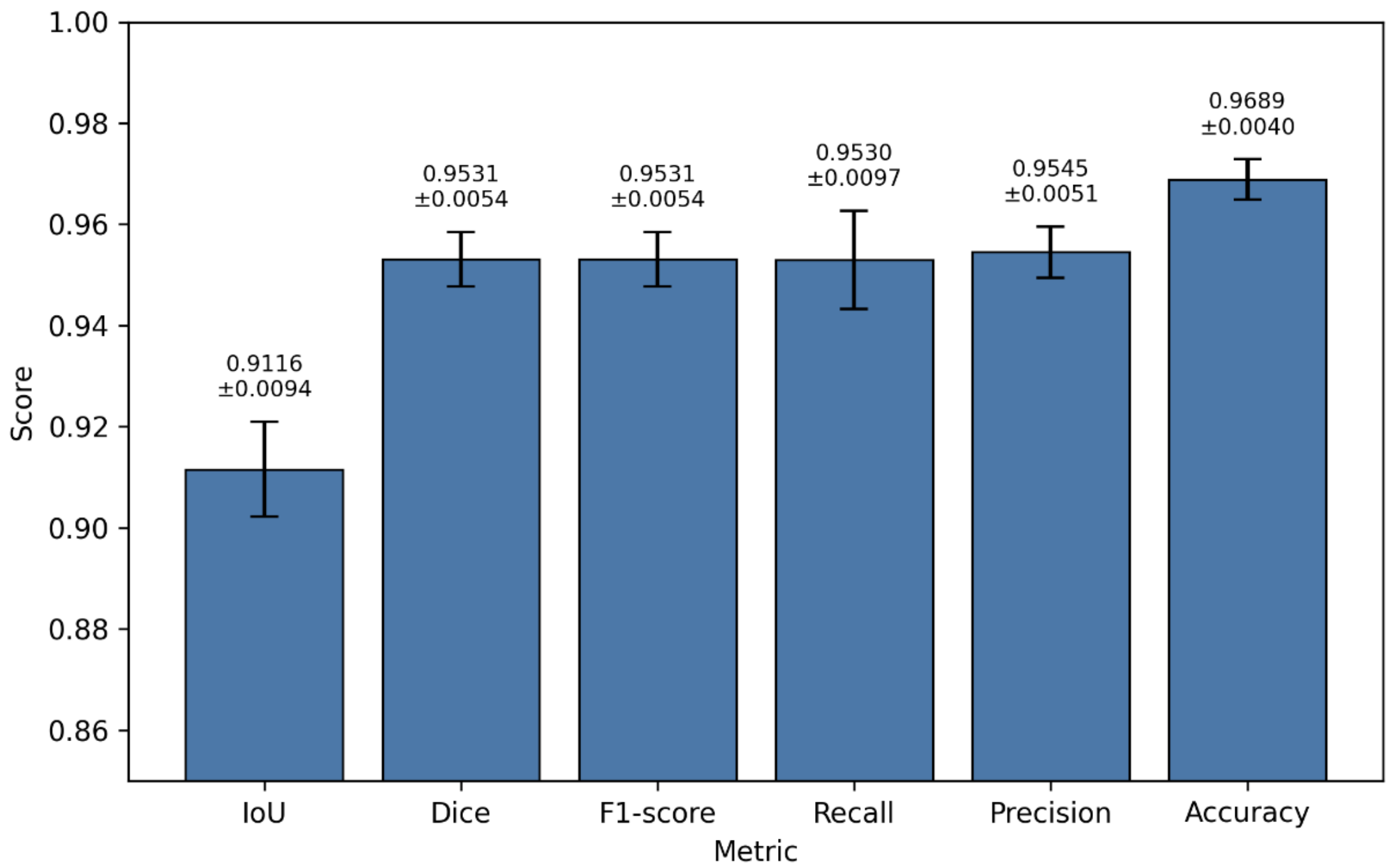

Figure.S30 Segmentation Performance Obtained by Five-Fold Cross-Validation

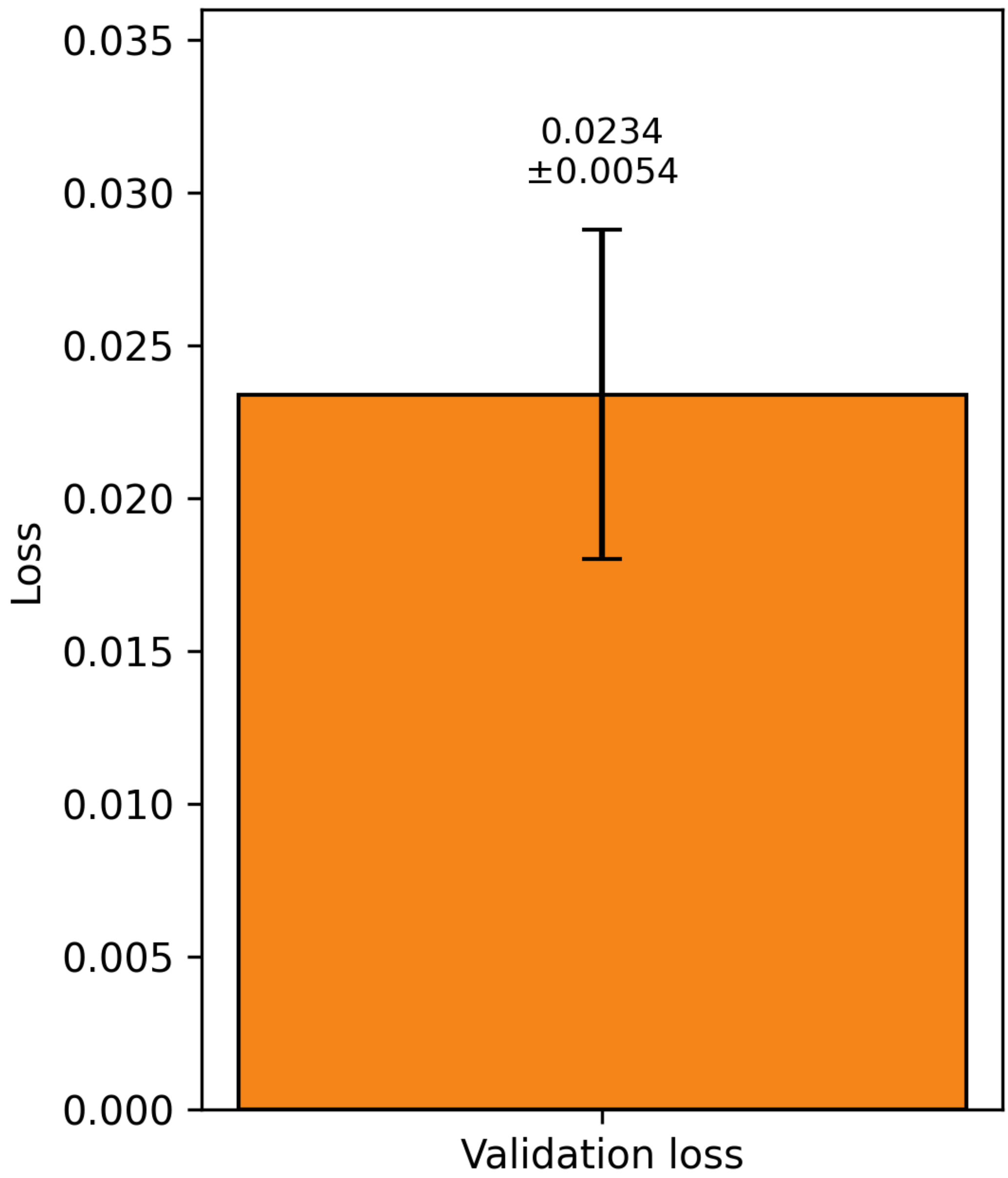

Figure.S31 Validation Loss Obtained by Five-Fold Cross-Validation

Furthermore, the average validation loss across the five folds was 0.0234 ± 0.0054, as shown in Figure S31. The consistently low validation loss, together with the minimal variation in metrics across folds, demonstrates that the optimized MU-KAN framework provides robust, reproducible, and high-precision segmentation performance suitable for nanoparticle analysis.

S8. Effect of input resolution on segmentation performance

To investigate the influence of input resolution on segmentation performance, an additional ablation study was conducted on the NanoSEM-464 dataset using four input resolutions (128 × 128, 256 × 256, 384 × 384, and 512 × 512). All training settings,

data augmentation strategies, and evaluation protocols were kept identical to those described in Section 2.2.3. Only the input image resolution was varied.

The network architecture, optimizer settings, learning rate schedule, batch size, and all other experimental configurations were strictly consistent with Section 2.2.3 to ensure a fair comparison across different resolutions.

Table S7.Effect of input resolution on segmentation performance on the NanoSEM-464 dataset.

Resolution	IoU	Loss	F1	Accuracy	Precision
128×128	0.9034	0.1341	0.9478	0.9663	0.9515
256×256	**0.9296**	**0.1031**	**0.9630**	0.9712	0.9604
384×384	0.9277	0.1095	0.9619	**0.9741**	**0.9630**
512×512	0.9271	0.1096	0.9616	0.9739	0.9583

As shown in Table S7, increasing the input resolution from 128 × 128 to 256 × 256 significantly improves segmentation performance, indicating that higher resolution preserves more nanoparticle boundary and morphological details. Further increasing the resolution to 384 × 384 and 512 × 512 yields only marginal performance changes, while introducing higher computational cost. Among all tested settings, 256 × 256 achieves the best overall performance in terms of IoU, F1-score, and loss. Therefore, 256 × 256 was selected as the default input resolution in this study.

S9. Comparison of OCR Methods for Scale Annotation Recognition

To justify the selection of Baidu OCR for scale annotation parsing, an additional comparison was conducted using the 370-image scale-bar dataset employed in this study. Three representative OCR methods were evaluated: Tesseract OCR, EasyOCR, and Baidu OCR.

For each image, the OCR system was required to recognize the numerical scale value and its corresponding unit (e.g., nm and μm) from the scale annotation. The OCR output was considered correct only when both the numerical value and the unit were correctly recognized. To ensure fair comparison, text normalization was performed prior to evaluation, including whitespace removal and unification of equivalent unit representations (e.g., μm, µm, and um).

Table S8. Recognition accuracy of different OCR methods on the 370-image scale-bar dataset.

OCR	Accuracy
Tesseract	45.95%
EasyOCR	64.32%
Baidu OCR	98.64%

As shown in Table S8, Baidu OCR achieved the highest recognition accuracy

among all evaluated methods. Compared with EasyOCR and Tesseract OCR, Baidu OCR demonstrated substantially more reliable recognition of SEM scale annotations containing numerical values and measurement units under varying image contrast conditions and annotation styles.

The superior performance of Baidu OCR can be attributed to its mature industrial deployment and optimization for mixed numerical-text recognition tasks. Since OCR is not the methodological focus of this study, Baidu OCR was adopted as an off-the-shelf auxiliary component for scale annotation parsing rather than as a research contribution.

These results support the use of Baidu OCR within the proposed scale recovery pipeline and justify its selection over the other evaluated OCR alternatives.

S10. Boundary and Metrology Accuracy Analysis

To quantitatively evaluate the statement that the proposed framework produces more coherent particle boundaries and more reliable particle measurements, an additional boundary and metrology analysis was conducted. Three complementary metrics were considered: Boundary F1-score, equivalent circular diameter mean relative error (Diameter MRE), and circularity mean relative error (Circularity MRE).

Boundary coherence was quantified using the Boundary F1-score, which evaluates the agreement between predicted and ground-truth particle boundaries. Let TP_b, FP_b and FN_b denote the numbers of true-positive, false-positive, and false-negative boundary pixels, respectively. Boundary precision and recall are defined as:

$$\text{Precision}_b=\frac{TP_b}{TP_b+FP_b} \tag{8}$$

$$\text{Recall}_b=\frac{TP_b}{TP_b+FN_b} \tag{9}$$

$$\text{Boundary F1}=\frac{2\times\text{Precision}_b\times\text{Recall}_b}{\text{Precision}_b+\text{Recall}_b} \tag{10}$$

Where TP_b, FP_b and FN_b denote the numbers of true-positive, false-positive, and false-negative boundary pixels, respectively.

A higher Boundary F1-score indicates better agreement between predicted and ground-truth particle boundaries.

For each matched particle pair, the equivalent circular diameter was calculated from the particle area:

$$D=2\sqrt{A/\pi} \tag{11}$$

where A denotes the particle area.

The mean relative error (MRE) of equivalent circular diameter was calculated as:

$$MRE_D=\frac{1}{N}\sum_{i=1}^{N}\frac{|D_i^{pred}-D_i^{GT}|}{D_i^{GT}}\times100\% \tag{12}$$

where D_i^{pred} and D_i^{GT} denote the equivalent circular diameters obtained from the predicted mask and the ground-truth mask, respectively, and N is the number of matched particle pairs.

Particle circularity was calculated as:

$$C = 4\pi \cdot A/P^2 \times 100\% \tag{13}$$

where A and P denote the particle area and perimeter, respectively.

The mean relative error of circularity was calculated as:

$$MRE_C = \frac{1}{N}\sum_{i=1}^{N}\frac{|C_i^{pred} - C_i^{GT}|}{C_i^{GT}} \times 100\% \tag{14}$$

where C_i^{pred} and C_i^{GT} denote the circularity values obtained from the predicted mask and the ground-truth mask, respectively.

Quantitative Comparison

Table S9 summarizes the Boundary F1-score, Diameter MRE, and Circularity MRE obtained by different segmentation methods on the NanoSEM-464 val set.

Table S9. Quantitative comparison of boundary and metrology accuracy.

Method	Boundary F1	Diameter MRE	Circularity MRE
BiSeNetV2	0.2025	32.9973	58.7808
U-Net(Dense)	0.6118	19.4789	21.3880
DCSAU-Net	0.7270	11.9569	15.1584
U-Net(ResUNet)	0.5904	24.9786	24.7676
U-Net(VGG)	0.6102	19.2338	20.0630
FPN	0.6859	20.3664	23.6001
deeplabv3plus	0.6878	21.6539	26.2188
Transunet	0.7134	15.8791	18.8603
U-KAN	0.8711	10.2200	13.5772
MU-KAN(ours)	0.9128	4.3165	6.7470

As shown in Table S9, MU-KAN achieved the highest Boundary F1-score among all evaluated methods, indicating the best agreement between predicted and ground-truth particle boundaries. Furthermore, MU-KAN achieved the lowest Diameter MRE and Circularity MRE, demonstrating the highest accuracy in particle-size and particle-shape estimation.

These results provide quantitative evidence that the proposed MU-KAN framework produces more accurate particle boundaries and more reliable downstream metrology measurements than the compared methods.

References

[1] Y. Li, Z. Wang, X. Zhang, U-KAN makes strong backbone for medical image segmentation and generation, arXiv preprint, 2024, arXiv:2406.02918.

[2] L. Bai, X. Ma, J. Liu, X. Sun, D. Zhao, D. G. Evans, Rapid Separation and Purification of Nanoparticles in Organic Density Gradients, J. Am. Chem. Soc., 2010, 132, 2333-2337.

[3] P. Pulcova, Nanoparticles_app: Nanoparticle Image Analysis Application, GitHub repository, available at: https://github.com/pulcovamon/Nanoparticles_app, accessed May 2026.
